# In-silico studies to recognize repurposing therapeutics toward arginase-I inhibitors as a potential onco-immunomodulators

**DOI:** 10.3389/fphar.2023.1129997

**Published:** 2023-04-18

**Authors:** Magdi E. A. Zaki, Sami A. Al-Hussain, Aamal A. Al-Mutairi, Abdul Samad, Arabinda Ghosh, Somdatta Chaudhari, Pravin N. Khatale, Prashant Ajmire, Rahul D. Jawarkar

**Affiliations:** ^1^ Department of Chemistry, Faculty of Science, Imam Mohammad Ibn Saud Islamic University, Riyadh, Saudi Arabia; ^2^ Department of Pharmaceutical Chemistry, Faculty of Pharmacy, Tishk International University, Erbil, Kurdistan Region, Iraq; ^3^ Microbiology Division, Department of Botany, Gauhati University, Guwahati, India; ^4^ Department of Pharmaceutical Chemistry, Progressive Education Society’s Modern College of Pharmacy, Pune, India; ^5^ Department of Medicinal Chemistry, Dr Rajendra Gode Institute of Pharmacy, Amravati, Maharashtra, India

**Keywords:** immunomodulatory, arginase-i, QSAR, virtual screening, MD simulation

## Abstract

Rudolf Virchow was the first person to point out the important link between immune function and cancer. He did this by noticing that leukocytes were often found in tumors. Overexpression of arginase 1 (ARG1) and inducible nitric oxide synthase (iNOS) in myeloid-derived suppressor cells (MDSCs) and tumour-associated macrophages (TAMs) depletes both intracellular and extracellular arginine. TCR signalling is slowed as a result, and the same types of cells produce reactive oxygen and nitrogen species (ROS and RNS), which aggravates the situation. Human arginase I is a double-stranded manganese metalloenzyme that helps L-arginine break down into L-ornithine and urea. Thus, a quantitative structure-activity relationship (QSAR) analysis was performed to unearth the unrecognised structural aspects crucial for arginase-I inhibition. In this work, a balanced QSAR model with good prediction performance and clear mechanistic interpretation was developed using a dataset of 149 molecules encompassing a broad range of structural scaffolds and compositions. The model was made to meet OECD standards, and all of its validation parameters have values that are higher than the minimum requirements (R^2^_tr_ = 0.89, Q^2^_LMO_ = 0.86, and R^2^_ex_ = 0.85). The present QSAR study linked structural factors to arginase-I inhibitory action, including the proximity of lipophilic atoms to the molecule’s centre of mass (within 3A), the position of the donor to the ring nitrogen (exactly 3 bonds away), and the surface area ratio. As OAT-1746 and two others are the only arginase-I inhibitors in development at the time, we have performed a QSAR-based virtual screening with 1650 FDA compounds taken from the zinc database. In this screening, 112 potential hit compounds were found to have a PIC50 value of less than 10 nm against the arginase-I receptor. The created QSAR model’s application domain was evaluated in relation to the most active hit molecules identified using QSAR-based virtual screening, utilising a training set of 149 compounds and a prediction set of 112 hit molecules. As shown in the Williams plot, the top hit molecule, ZINC000252286875, has a low leverage value of HAT i/i h* = 0.140, placing it towards the boundary of the usable range. Furthermore, one of 112 hit molecules with a docking score of −10.891 kcal/mol (_P_IC_50_ = 10.023 M) was isolated from a study of arginase-I using molecular docking. Protonated ZINC000252286875-linked arginase-1 showed 2.9 RMSD, whereas non-protonated had 1.8. RMSD plots illustrate protein stability in protonated and non-protonated ZINC000252286875-bound states. Protonated-ZINC000252286875-bound proteins contain 25 Rg. The non-protonated protein-ligand combination exhibits a 25.2-Rg, indicating compactness. Protonated and non-protonated ZINC000252286875 stabilised protein targets in binding cavities posthumously. Significant root mean square fluctuations (RMSF) were seen in the arginase-1 protein at a small number of residues for a time function of 500 ns in both the protonated and unprotonated states. Protonated and non-protonated ligands interacted with proteins throughout the simulation. ZINC000252286875 bound Lys64, Asp124, Ala171, Arg222, Asp232, and Gly250. Aspartic acid residue 232 exhibited 200% ionic contact. 500-ns simulations-maintained ions. Salt bridges for ZINC000252286875 aided docking. ZINC000252286875 created six ionic bonds with Lys68, Asp117, His126, Ala171, Lys224, and Asp232 residues. Asp117, His126, and Lys224 showed 200% ionic interactions. In protonated and deprotonated states, GbindvdW, GbindLipo, and GbindCoulomb energies played crucial role. Moreover, ZINC000252286875 meets all of the ADMET standards to serve as a drug. As a result, the current analyses were successful in locating a novel and potent hit molecule that inhibits arginase-I effectively at nanomolar concentrations. The results of this investigation can be used to develop brand-new arginase I inhibitors as an alternative immune-modulating cancer therapy.

## Graphical Abstract

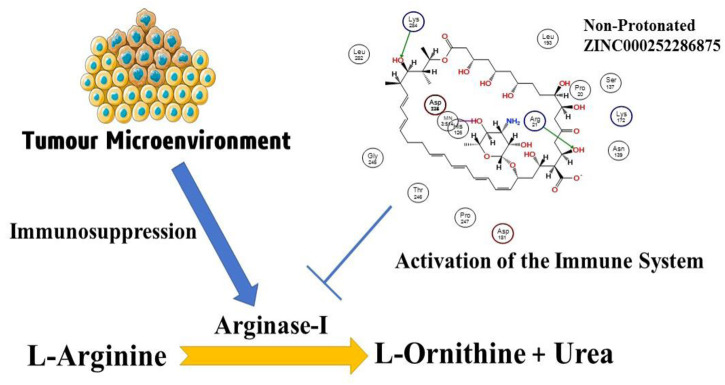

## Introduction

Immunotherapy for cancer has become a successful treatment modality. Checkpoint inhibitors disable immune response-regulating pathways to treat several solid tumours (Sosnowska et al., 2021). For a century, researchers have tried to use the immune system to fight cancer. Recently discovered molecular processes help tumour elude anti-tumor immunity (O’Donnell et al., 2018). Amino acid metabolism controls immune response. Increased amino acid degradation hinders T cell activation, proliferation, and effector function (Murray, 2016). Modulating amino acid metabolism is promise in several immunotherapies since it controls the immune response. Essential amino acids inhibit T cell activation and growth. Cancer upregulates only L-arginine-degrading arginases and tryptophan hydrolyzing enzymes (Grzywa et al., 2020). In 1904, Kossel and Dankin discovered arginase and found it in all species. ARG-1 and ARG-2 are manganese-containing arginase enzymes and share 58% amino acid homology and 100% in the active sites (Yang and Ming, 2013). Trimers of mammalian arginases are the active form (Cama et al., 2003; Cama et al., 2004). Although they both hydrolyze L-arginine into L-ornithine and urea, their cellular expression, regulation, and subcellular localization are distinct (Jenkinson et al., 1996). A high level of arginase (Arg1 or Arg2) expression is associated with a dismal prognosis in several cancers, including lung (Miret et al., 2019), head and neck (Bron et al., 2013), neuroblastoma (Mussai et al., 2015), acute myeloid leukaemia (Mussai et al., 2013), pancreatic ductal carcinoma (Algül et al., 2013), ovarian (Czystowska-Kuzmicz et al., 2019), and colorectal malignancies (Ma et al., 2019). However, the clear impact of increased Arg activity on patients’ prognoses has not been reported in these tumour types. In addition, increased Arg activity was found in the skin (Gökmen et al., 2001), cervical, thyroid (follicular, papillary, and follicular variant of papillary) (Cerutti et al., 2004), gastric (Akiba et al., 2016), bile duct, hepatocellular (Obiorah et al., 2019), breast (Porembska et al., 2003) cancer (Gabitass et al., 2011). In addition to melanoma (Ascierto et al., 2005; Gabitass et al., 2011) and renal carcinoma (Yoon et al., 2007), (Ochocki et al., 2018), which are both Arg auxotrophic malignancies, no associations between Arg concentrations and survival have been reported.

**FIGURE 1:**
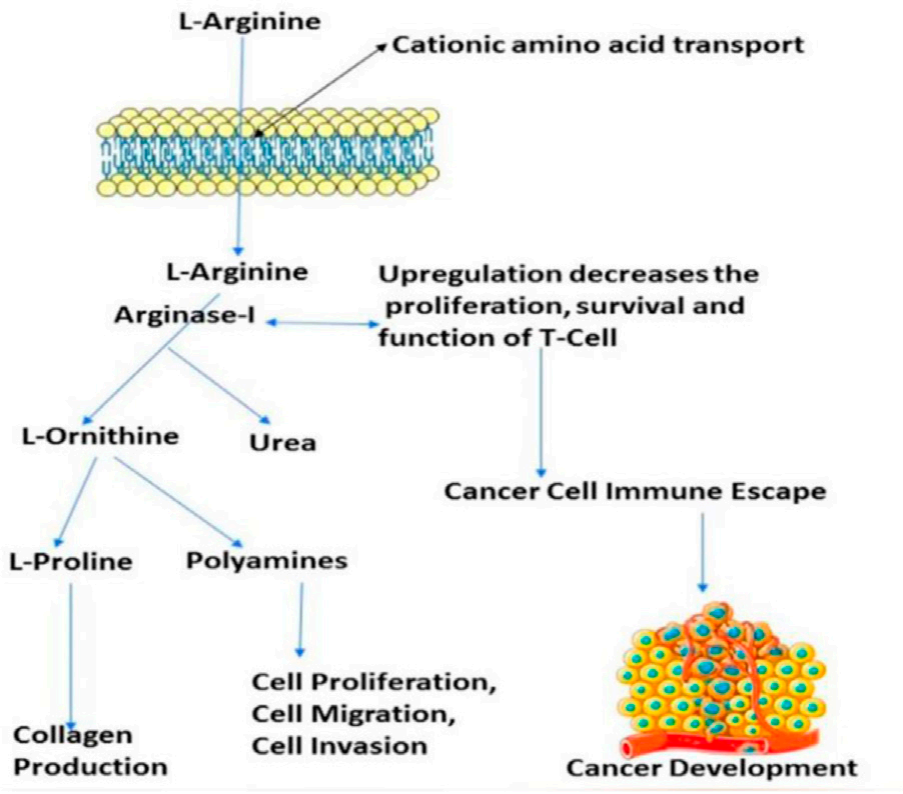
Presentation of the involvement of arginase-I in the cancer development, and immune mechanism.

The number of studies discussing the role of arginase in the immune system has exploded in the last few years. This is due to the enzyme’s importance in a variety of inflammation. Researchers discovered that arginase is responsible for or involved in regulating a wide range of physiological processes, including inflammation-dysfunctional immunological response, tumour immune evasion, inflammation, fibrosis, and immunopathology of serious infection illnesses (Bronte and Zanovello, 2005). L-arginine is broken down by the arginase enzyme into urea and L-ornithine (see Figure 1). While both types of arginase serve as catalysts, their cellular expression, regulation, metabolic response, and intracellular location are distinct (Jenkinson et al., 1996). One of the liver’s main sources of expression for the urea cycle is the L-arginine urea hydrolase, AI (EC 3.5.3.1), and isoform arginase I detoxifies ammonia. The cycle is distributed across two cellular compartments. The (mitochondrion/cytosol) protein uses arginine as a cytosolic enzyme (Jenkinson et al., 1996). Rat liver arginine crystallised as a trimeric enzyme with a cleft at the catalytic site and a binuclear manganese cluster (Kanyo et al., 1996). Arginase I, a 322-amino-acid protein from humans, and Arginase II have 58% of the same amino acid sequence. The human arginase I gene was found to be placed on chromosome 6q23 more than 20 years ago, if not more (Dizikes et al., 1986) (Haraguchi et al., 1987).

Arginase overexpression in immune cells (T-cells) may contribute to disease pathogenesis because it reduces NO-mediated cytotoxicity by consuming L-arginine, boosts collagen production and fibrosis by creating proline, and promotes cellular proliferation by generating polyamines. Arginase is crucial to tumour immunology, according to years of research (Sica and Bronte, 2007); (Rodríguez and Ochoa, 2008). Earlier research have focused on arginase expression in primitive tumours’ from mice or humans, carcinogenic tissue, and cell culture (Wu et al., 1996) and how it may promote tumour development, polyamine synthesis, or NO-mediated tumour cytotoxicity.

Arginase has been found in a number of human tissues and bodily fluids, including post-injury PBMCs (Ochoa et al., 2001), inflammatory synovial fluid macrophages (due to arginase II) of patients with arthritis, and inflammatory cells in bronchoalveolar lavage fluid of patients with asthma (Zimmermann et al., 2003) (Zea et al., 2006). Only PMN, which are found only in normal blood donors’ peripherally circulating human leukocytes, are capable of expressing arginase (Munder, 2005). It was proven that the enzyme is constitutively present in the azurophil granules of human PMN by using biochemical fractionation and immunoelectron microscopy. Thus, the enzyme provides a unique oxygen-independent anti-microbial defence mechanism (Munder, 2005).

Various publications reported N-hydroxy-nor-arginine (nor-NOHA), a micromolar inhibitor of arginase, which was derived from the NO production intermediate N-hydroxy-L-arginine (NOHA). Subsequently, two more molecules, 2(S)-amino-6-boronohexanoic acid (ABH) and S-(2-boronoethyl)-L-cysteine (40, BEC), are currently used as standards for arginase inhibition since they were inspired by borate’s established role in manganese and arginase binding (Pudlo et al., 2017). Unfortunately, there is a shortfall of drug-like hARGI inhibitors; to date, only a small number of inhibitor families have been explored against this protein’s action (Pudlo et al., 2017). The theoretical effort only analysed experimental structural data from many drugs’ crystal structures in the hARGI and hARG binding sites. Hence, *in silico* methods like QSAR help uncover a new arginase inhibitor by discovering a molecule’s unrecognised arginase-inhibiting characteristics. Low-cost computational methods like QSAR (3D-quantitative structure-activity relationships), protein-ligand molecular docking, QSAR-based virtual screening, MD simulation, molecular mechanics generalised borne surface area, and others can process experimental data and provide useful information about compound properties that affect their activities. So, this study uses QSAR analysis of 149 Arginase I inhibitors with correct experimental half-minimal inhibitory concentrations (IC_50_) and virtual screening to find a new target. The results may assist to develop an arginase I inhibitor.

## Materials and methods

### Data collection and curation

In this investigation, a QSAR evaluation was performed using a curated dataset of 149 Arginase I inhibitors with correct experimental half-minimum inhibitory doses (IC_50_) reported in nM units that were retrieved from the binding database (https://www.binding.org/bind/chemsearch, accessed on 2 May 2022) (Liu et al., 2007). This data set includes not only a wide chemical space comprised of compounds with a variety of pharmacophoric features, but also a very unique range of bioactivity values provided in IC_50_ and covering the range 0.1-1176000 nM (See Supplementary Table S1).

**FIGURE 2:**
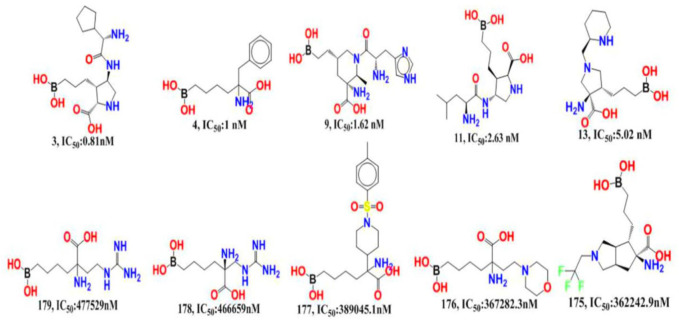
Depiction of the chemical structures for the five most active and five least active Arginase I inhibitors from the present dataset.

The experimentally reported IC_50_ values in nanomolar units were converted to equivalent molar units and then converted to pIC_50_ values using the formula pIC_50_ = -logIC_50_ for statistical purposes. Figure 2 is a representation of the five most active and five least active substances in the dataset.

### Molecular descriptor calculation and objective feature selection (OFS)

The 3D structures of all the molecules in the current dataset are generated and geometrically optimised using the MMFF94 force field (O'Boyle et al., 2011; Tosco et al., 2011). These geometry-optimized molecules were then run *via* PyDescriptor, a plugin for the molecular modelling programme PyMOL that has a database of over 40,000 chemical descriptors, ranging in dimension from 1D to 3D (Masand and Rastija, 2017). Data pruning is necessary when dealing with such a vast number of molecular descriptors. To accomplish the goal, an objective feature selection (OFS) in QSARINS v2.2.4 was employed (Gramatica et al., 2013). A limited pool of 694 distinct molecular descriptors were provided by the OFS technique, after excluding out near-constant, constant, or highly correlated (|R|> 0.90) molecular descriptors (The computed characterizations are included in Supplementary Table S2).

### Splitting of the data set molecules into training and external sets and subjective feature selection

The entire dataset was arbitrarily split into a training set of 119 molecules (an 80%) used to develop a QSAR model and a prediction set of 30 molecules (an 20%) used to rigorously validate the developed QSAR models for reliability and predictiveness using QSARINS v2.2.4’s random splitting feature. Methods for subjective feature selection using genetic algorithm-reinforced multi-linear regression (GA-MLR) have been implemented in QSARINS v2.2.4 using Q^2^_LOO_ as the fitness parameter (SFS) (Gramatica et al., 2012; Gramatica et al., 2013). The created QSAR models are put to the test by using a range of different validation criteria, including the coefficient of determination (*r* ^2^), leave-one-out (Q^2^_LOO_), and leave-many-out (Q^2^_LMO_), all of which have been documented in the literature. Reducing the intercorrelation between the descriptors is facilitated by a QUIK (Q under the influence of K) value of 0.05. The data-fitting hypothesis is tested at 2,000 iterations of Y randomization by computing correlation coefficients (Dearden et al., 2009). The predictiveness of the QSAR model is measured by how well the predicted value matches the anticipated or experimental value, and it may decrease even in the presence of a single outlier. We have thus made an effort to draw attention to the outlier using these molecules, which validated a noticeably high residual value in GA-MLR QSAR models. Additionally, by contrasting the predicted value with the standard residual values, we were able to identify outlier compounds. Similar structural variations were found in database compounds using the Williams plot’s leverage effect. It is possible to identify the application domain of the advanced QSAR model by combining the leverage and the typical residuals.

### Building regression model and its validation

A good QSAR model that has been correctly verified using various approaches such as cross-validation, external validation, Y-randomization, and the applicability domain (Williams’s plot) is useful for future applications in virtual screening, molecular optimization, decision-making, and so on. The statistical parameters mentioned below are typically used to verify a model, along with their suggested threshold values (Martin et al., 2012; Masand et al., 2014; Fujita and Winkler, 2016; Gramatica, 2020). The formulas for obtaining these statistical characteristics are presented in the (Supplementary Table S3). Williams’ plots were also used to evaluate the QSAR model’s applicability domain (Consonni et al., 2009; Chirico and Gramatica, 2011; Consonni et al., 2019). A genetic functional algorithm in conjunction with multiple linear regression was used to develop a robust and accurately validated QSAR model, which provided a deep understanding of the understated and hidden pharmacophoric features that control certain biological activity and lend a sufficient external predictive capability. As a consequence, a novel approach was used, in which several models were built using 80% of the training set and verified using random splitting on the remaining set (20% prediction set). As a consequence, a QSAR model based on six descriptors was built and tested on a prediction set (which was initially the training set), and the best predicted model is reported for analysis.

### QSAR based virtual screening

A database of 1615 FDA molecules was obtained from the Zinc database for QSAR-based VS. Before calculating molecular descriptors, 3D-structures of molecules were generated in the same way that the modelling set was. Based on the estimated chemical descriptors, a well-validated six-parametric division set QSAR model was used to predict the arginase-I inhibitory activity of 1615 FDA molecules obtained from the zinc database (Jawarkar et al., 2022a; Bakal et al., 2022; Jawarkar et al., 2022b; Ghosh et al., 2022).

### Target preparation

Arginase-I (PDB ID: 3kv2), the primary protease of interest, was retrieved from the Protein Data Bank’s structural database (https://www.rcsb.org/structure/3kv2) and imported into a molecular editor with an open-source licence (Discovery Studio Visualizer 4.0). The UCSF Chimera employed the steepest descent approach to identify 1,000 steps and then the conjugate gradient of energy minimization strategy to optimise the structure of the hit (FDA) molecules, which were retrieved from a ZINC database after QSAR-based virtual screening.

### Molecular docking analysis

The primary arginase I pdb file was retrieved from the Protein Data Bank’s structural database (https://www.rcsb.org/structure/3kv2; viewed on 7 May 2022). Based on X-ray resolution and sequence completeness, pdb 3kv2 was selected. Ramachandran’s plot was prepared before actual docking simulations to guarantee the protein’s health. The improved protein was subjected to docking analysis. Although all of the compounds were docked in the active site, for the sake of brevity, just the docking stance of the most active molecule, number 4, is detailed here. For molecular docking analysis, the NRG Suite software was employed. This open-source programme is available as a PyMOL plugin (www.pymol.org). FlexAID may be used in docking simulations to identify protein surface cavities and target binding locations (Gaudreault et al., 2015). It employs genetic algorithms to retrieve conformational information and simulates ligand and side chain flexibility and covalent docking. To get the best performance with NRGSuite, we used a flexible rigid docking technique with the following default parameters: Compliance of Ligands Ligand is denoted by a reference number. No limit exists. Spherical binding site input method, 0.385 3D grid spacing, and side chain rigidity. A reference number is used to reflect Ligands’ adaptability. No limit exists. Water molecules are encased by the HET group. 0.1% was van der Wall’s magnetic permeability. And there is no indication of the solvent type; number of chromosomes: 1,000; number of generations: 1,000; fitness model: share; reproduction model: population explosion; number of TOP complexes: five. For the purpose of validating molecular docking, a known peptidomimetic inhibitor of Mpro was used to validate the docking methodology.

### Molecular dynamics simulation (MD-Simulation) and free energy landscape (FEL) analysis

The MD simulations were carried out in triplicate using the Desmond 2020.1 from Schrödinger, LLC, on dock complexes for arginase-I (PDB I.D.: 3kv2) and ZINC000252286877. To ensure that the results were repeatable, duplicate samplings were performed with the same parameters for each MD run. This system utilises the OPLS-2005 force field (Shivakumar et al., 2010) and an explicit solvent model with SPCwater molecules. To neutralise the charge, Na+ ions were added. To imitate the physiological environment, 0.15 M NaCl solutions were introduced to the system. To retrain the system over the protein-ZINC000252286877 complex, the system was first equilibrated using an NVT ensemble for 500 ns Following the preceding phase, an NPT ensemble was used to execute a short equilibration and minimization run for 12 ns In all simulations, the NPT ensemble was set up using the Nose-Hoover chain coupling scheme (Jorgensen et al., 1983), with a temperature of 27°C, a relaxation duration of 1.0 ps, and a pressure of 1 bar. A 2 FS time step was chosen. With a relaxation duration of 2 ps, the Martyna–Tuckerman–Klein chain coupling scheme (Martyna et al., 1992) barostat method was employed for pressure control. Long-range electrostatic interactions were calculated using the particle mesh Ewald method (Toukmaji and Board, 1996), with the Coulomb interaction radius set at 9. The bonded forces were calculated using the RESPA integrator with a time step of 2 fs for each trajectory. To check the stability of the MD simulations, the root mean square deviation (RMSD), radius of gyration (Rg), root mean square fluctuation (RMSF), and quantity of hydrogen (H-bonds) were computed. Geo measures v 0.872 was used to calculate the free energy landscape of protein folding on a chemically 4-bound complex (Kagami et al., 2020). The MD trajectory versus RMSD and Radius of Gyration (Rg) energy profile of folding was recorded in a 3D plot using the matplotlib python package utilising Geo measures, which includes a sophisticated library of g_sham (Ghosh et al., 2022).

### Molecular mechanics generalized born and surface area (MMGBSA) calculations

Using the premier molecular mechanics generalised Born surface area (MM-GBSA) module, the binding free energy (Gbind) of docked complexes was determined during MD simulations of arginase I complexed with ZINC000252286877. (Schrodinger Suite, LLC, New York, NY, 2017-4). OPLS 2005 force field, VSGB solvent model, and rotamer search methods were used to compute the binding free energy. The MD trajectory frames were chosen at 10-ns intervals after the MD run. Equation 1 was used to compute the total free energy of binding.:

$$∆Gbind=Gcomplex\;–\;\left(Gprotein+Gligand\right)$$

Where, **∆Gbind** = binding free energy, **Gcomplex** = free energy of the complex, **Gprotein** = free energy of the target protein, and **Gligand** = free energy of the ligand.

The MMGBSA result trajectories were further examined for post-dynamic structure modifications.

## Results

As mentioned in the introduction, the goal was to create a GA-MLR model with a combination of mechanistic explanations and high predictive power. In the present analysis, we have uncovered a lot of structural traits. The following are the parameters of the newly created six-parameter model and their statistical validation.

### QSAR model

_**P**_IC_50_ = −7.008 (±2.115) + 19.791 (±3.496) * rsa + 0.344 (±0.067) * com_lipohyd_3A + 0.905 (±0.125) * fringNdon3B + 0.402 (±0.16) * fsp2OC9B + −0.375 (±0.069) * fHringC2B + −0.567 (±0.205) * fringCC3B +.

### Statistical parameters associated with model

*R* ^2^: 0.8926, R^2^_adj_: 0.8867, R^2^-R^2^_adj_:0.0059, LOF:0.3073, Kxx: 0.3352, Delta K: 0741, RMSE tr:0.4970, MAEtr:0.4178, RSStr: 28.6575,CCCtr:0.9432, s: 0.5128, F: 150.9529, Q^2^_loo_: 0.8779,R^2^-Q^2^_loo_: 0.0146, RMSE_cv_: 0.5298, MAE_cv_: 0.4450, PRESS_cv_: 32.5651, CCC_cv_: 0.9356, Q^2^_LMO_: 0.8761, R^2^_Yscr_: 0.0515, Q^2^_Yscr_: −0.0752, RMSE AV Yscr: 1.4768, RMSE_ext_: 0.5899, MAE_ext_: 0.4993,PRESS_ext_: 9.7450, R^2^_ext_: 0.8585, Q^2^-_F1_: 0.8503, Q^2^-_F2_: 0.8486, Q^2^-_F3_: 0.8487, CCC_ext_: 0.9260, r^2^m aver.: 0.7970, r^2^m delta: 0.0480, Calc. External data regr. Angle from diagonal: −1.5552°, Exp(x) vs. Pred(y): *R* ^2^: 0.8780, R′^2^o: 0.8646, k′: 0.9934, Clos’: 0.0152, r'^2^m: 0.7765, Pred(x) vs. Exp(y): *R* ^2^: 0.8780, R^2^o: 0.8779, k: 0.9997, Clos: 0.0001, r^2^m: 0.8696, Exp(x) vs. Pred(y): *R* ^2^: 0.8585, R′^2^o: 0.8566, k′: 0.9904, Clos’: 0.0022, r′^2^m: 0.8210, Pred(x) vs. Exp(y): *R* ^2^: 0.8585, R^2^o: 0.8486, k: 1.0007, Clos: 0.0115, r^2^m: 0.7730.

Numerous statistical parameters, including *R* ^2^ (coefficient of determination), R^2^_adj_. (adjusted coefficient of determination), R^2^_cv_ (Q^2^_LOO_) (cross-validated coefficient of determination for leave-one-out), R^2^_ex_ (external coefficient of determination), Q^2^-_Fn_, and CCC_ex_ (concordance correlation coefficient), etc., have high values, indicating that the developed QSAR model is statistically robust (mean absolute error).

**FIGURE 3:**
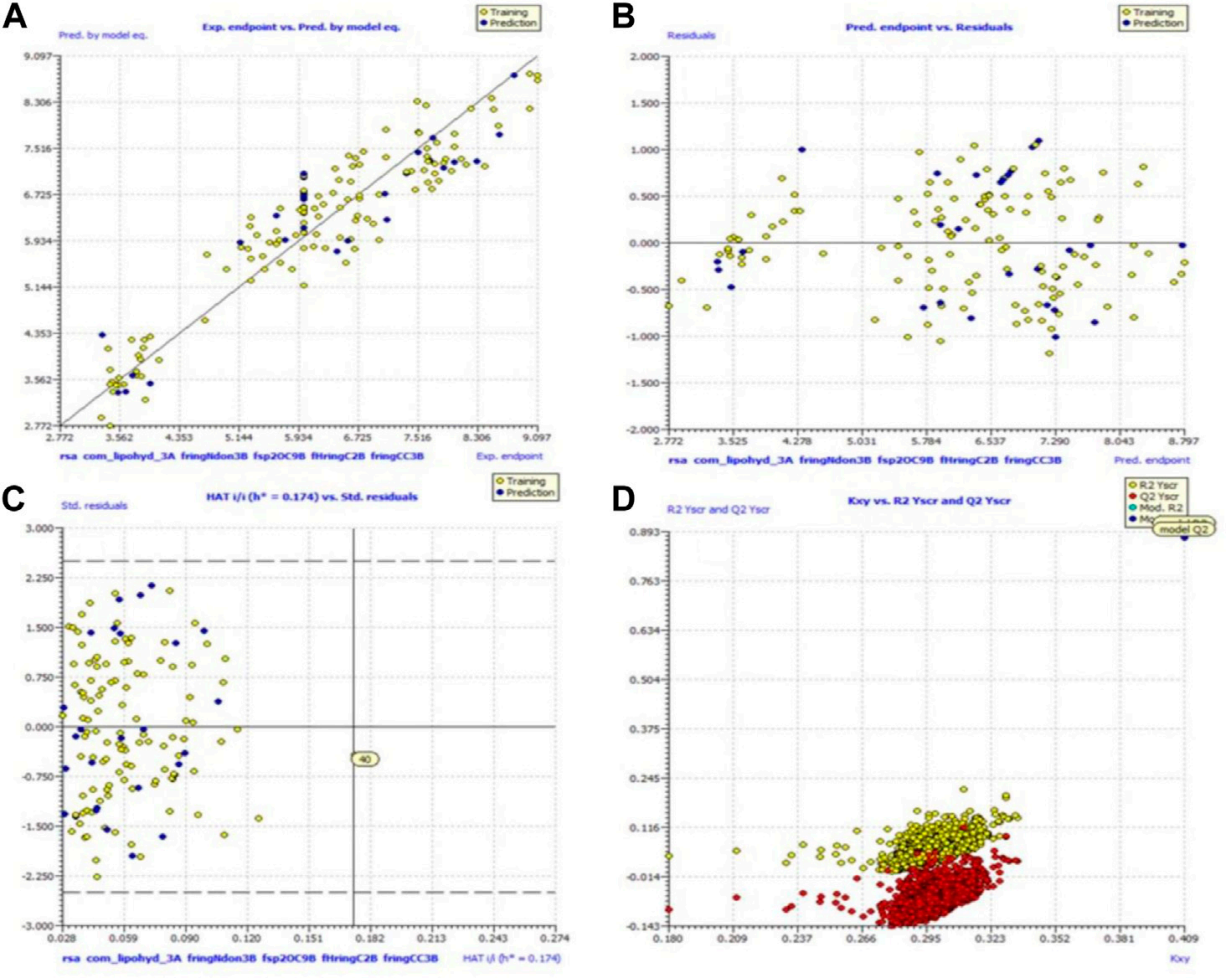
Different graphs related to model **(A)** experimental vs. predicted pKi (the solid line represents the regression line); **(B)** experimental vs. residuals; **(C)** William’s plot for applicability domain (the vertical solid line represents h* = 0.021 and horizontal dashed lines represent the upper and lower boundaries for applicability domain); **(D)** Y-randomization plot.

Because of this, the model has a high level of external prediction accuracy, no spurious correlations, and passes the required minimum values for its most critical parameters. In the Supplemental Materials, the necessary method for determining these parameters is provided. Williams plots were used to assess the range of this model’s applicability. Therefore, it satisfies all the criteria for creating a reliable QSAR model as proposed by the OECD. A variety of model-related graphs are shown in Figure 3.

There are six descriptors used in the QSAR model, which are listed in Table 1. Three of the descriptors, com_lipohyd_3A, fringNdon3B, and fsp2OC9B, all exhibit positive coefficients in the QSAR model, suggesting that boosting their values might improve the activity profile, whereas the other three, rsa, fHringC2B, and fringCC3B, all have negative coefficients (see Table 1). Different forms of pharmacophoric properties, which determine the inhibitory profile, are correlated with each molecular descriptor, which is a numerical representation of structural features. However, it should be remembered that the ultimate biological activity (IC_50_) of a molecule cannot be explained or decided by a single structural property alone. Combinations of diverse structural traits and as-yet-unknown components provide the IC_50_, biological activity, etc. Different properties either promote or inhibit the intended pharmacological effect. It is generally agreed that the biological activity of a compound is determined by the presence of two or more pharmacophoric groups (pharmacophore synergism).

**TABLE 1:** Different molecular descriptors present in the developed QSAR model and their description.

Molecular descriptor	Description
com_lipohyd_3A	Occurrence of lipophilic hydrogen atoms within 3A from the centre of mass of the molecule
fringNdon3B	Frequency of occurrence of donor atom exactly at 3 bonds from the ring nitrogen atom
fsp2OC9B	Frequency of occurrence of carbon atoms exactly at 9 bonds from the sp2 hybridized oxygen atoms
rsa	Ratio of molecular surface area to the solvent accessible surface area
fHringC2B	Frequency of occurrence of ring carbon atoms exactly at 2 bonds from the hydrogen atoms
fringCC3B	Frequency of occurrence of carbon atom exactly at 3 bonds from the ring carbon atoms

### Correlation matrix

An inter-correlation coefficient threshold of 0.95 was included in the current QSAR model to avoid overfitting. There is no association between the various descriptors used in the current QSAR model, according to the correlation matrix between the descriptors that is also shown in Table 2.

**TABLE 2:** Presentation of the correlation matrix for the descriptor used to developed QSAR Model.

	rsa	com_lipohyd_3A	fringNdon3B	fsp2OC9B	fHringC2B	fringCC3B
rsa	1					
com_lipohyd_3A	0.0015	1				
fringNdon3B	0.3549	−0.3973	1			
fsp2OC9B	0.3452	−0.3594	0.1067	1		
fHringC2B	−0.3015	−0.224	−0.1	0.2535	1	
fringCC3B	0.6016	0.0477	0.4928	0.189	−0.2484	1

## Discussion

Three of the six descriptors in the developed QSAR model displayed a positive coefficient, so increases in their value enhance the biological activity. Amongst these descriptors, *viz.*, fsp2OC9B, fHringC2B, and fRingCC3B, the kind of carbon atom, ring or non-ring, plays a significant role in determining the araginase I inhibitory activity. Nevertheless, it is important to keep in mind that the descriptors in the created QSAR model are intricately interrelated, meaning that improving the value of one descriptor might drastically alter the value of another descriptor. Given that molecular descriptors are a mathematical description of pharmacophores, this results in a significant alteration in a molecule’s biological profile and suggests pharmacophore synergism.

## Mechanistic interpretation

**FIGURE 4:**
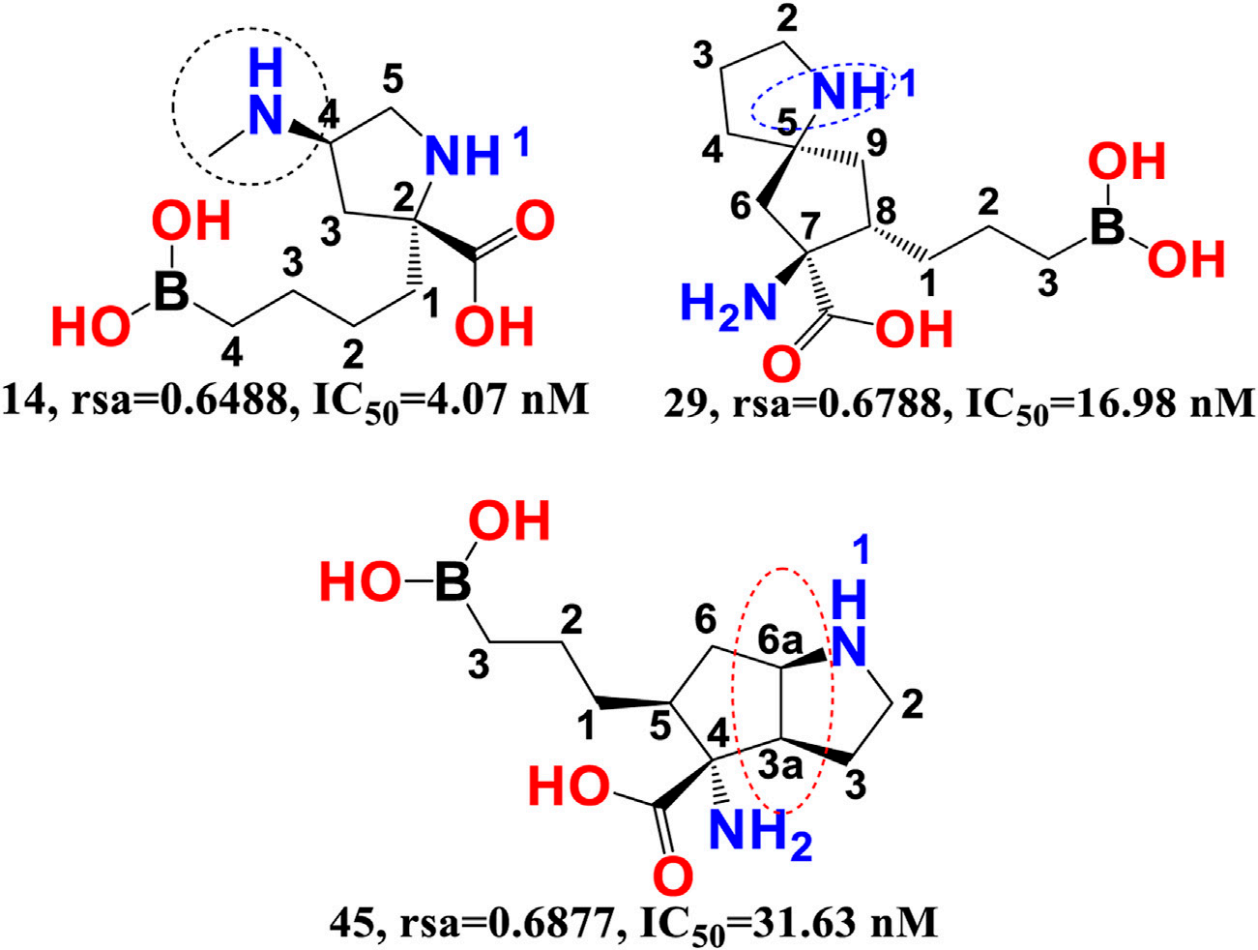
Illustration of the molecular descriptor rsa for the molecular pair 14, 29, and 45 only.

rsa-The chemical descriptor rsa (ratio of surface area) encodes information on the molecular surface area to solvent accessible surface area ratio and shows a negative correlation with araginase inhibitory efficacy. The inhibitory activity of araginase 1 is greatly affected by even a minor change in rsa. Because rsa is the ratio of the values of All_MSA and All_SASA, the big probable value of All_SASA to the tiny value of All_MSA will set rsa to the lower value, hence boosting the molecule’s araginase inhibitory activity (IC_50_). Comparing molecule 14 (IC_50_ = 4.07 nm, rsa = 0.6488, All_MSA = 265.8, All_ SASA = 442.03) to molecule 29 (_P_IC_50_ = 16.98 nm, rsa = 0.6788, All_MSA = 460.1, All_SASA = 677.8) and molecule 45 (IC_50_ = 31.63 nm, rsa = 0.6877, All_MSA = 446.01, All_ SASA = 648 support this observation (see Figure 4).

**FIGURE 5:**
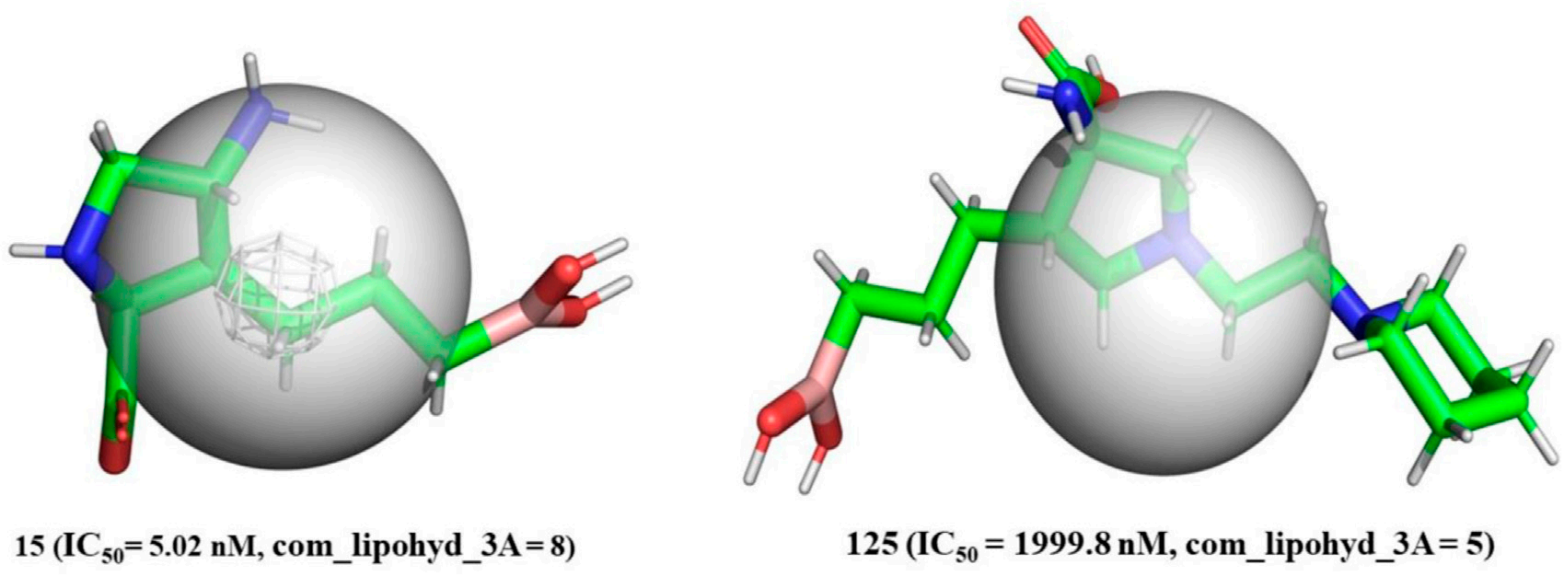
Depiction of com_Hhyd_3A using molecule 15 (IC_50_ = 5.02 nM, com_lipohyd_3A = 8) and molecule 125 (IC_50_ = 1999.8 nM, com_lipohyd_3A = 5) as representative examples only (Radius of gray sphere is 3 Å).

Moreover, variations in the number of carbon atoms have a significant effect on the polarity and lipophilicity of the molecule, as shown by molecule 14s ClogP of 0.600 and molecules 29 and 45s ClogP of −2.27 and −2.83, respectively. This finding suggests that even minute differences in the number of carbon atoms may alter the rsa, which has a major effect on both lipophilicity and polarity, thus highlighting the importance of a balance between polarity and lipophilicity in arginase inhibitory activity. Interestingly, the Christianson group at the University of Pennsylvania synthesised the first boron-containing arginase inhibitor, 2-(S)-amino-6-boronohexanoic acid, and noticed that since the active site of the araginase 1 enzyme contains several polar amino acid residues, the ligands must also be very polar. This observation aligns perfectly with the QSAR results. Consequently, QSAR findings demonstrated the relevance of polarity and lipophilicity in araginase 1 inhibitory action by revealing the same feature (Baggio et al., 1997).

com_lipohyd_3A-The com_lipohyd_3A molecular descriptor reflects the total amount of lipophilic hydrogen atoms with partial charges in the range ± 0.200 and located within 3 Å of the molecule’s center of mass (com). As the partial charge must be within the range of 0.200, this descriptor describes the function of non-polar hydrogens present within 3 of the molecule’s centre of mass (Todeschini and Consonni, 2009). In the constructed QSAR model, the descriptor com_lipohyd_3A has a positive coefficient, indicating that the higher the value of such lipophilic hydrogens, the greater will be their activity. This could be achieved by sustaining the lipophilic (non-polar hydrogen) atoms in future drug designs. This indirectly points out that the presence of lipophilic hydrogens, in turn, causes hydrophobic groups nearer the centre of mass of the molecule to be beneficial for increasing the IC_50_ value. Because hydrogen is a very small element in comparison to other elements and because replacing it with any other element would result in an increase in steric bulk, bulkiness near the centre of mass of the molecule is extremely unfavourable for increasing arginase inhibitory activity. In addition, the value of com_lipohyd_3A is determined by the location of the centre of mass, which shifts depending on where the various groups and atoms are located (positional isomers). Because of this, the value of com_lipohyd_3A varies depending on the positional isomer. Take, for example, molecules 15 (IC_50_ = 5.02 nM, com_lipohyd_3A = 8) and 125 (IC_50_ = 1999.8 nM, com_lipohyd_3A = 5) (depicted in Figure 5). Therefore, the descriptor does an excellent job of capturing the importance of positional isomerism in relation to the calculation of the _P_IC_50_ value. Thus, the descriptor com_lipohyd_3A and its positive connection (correlation coefficient R = 0.20) with _P_IC_50_ underline the critical role played by the presence of lipophilic groups, steric bulkiness close to the molecule’s centre of mass, and positional isomerism. This observation is further confirmed by comparing following pairs of molecules: 2 (IC_50_ = 0.85 nM, com_lipohyd_3A = 2) with 21 (IC_50_ = 10 nM, com_lipohyd_3A = 1), 13 (IC_50_ = 3.23 nM, com_lipohyd_3A = 4) with 28 (IC_50_ = 16.28 nM, com_lipohyd_3A = 3), 34 (IC_50_ = 20.41 nM, com_lipohyd_3A = 7) with 81 (IC_50_ = 346.7 nM, com_lipohyd_3A = 4), 53 (IC_50_ = 77.98 nM, com_lipohyd_3A = 3) vs. 122 (IC_50_ = 1,399.5 nM, com_lipohyd_3A = 1), 16 (IC_50_ = 6.02 nM, com_lipohyd_3A = 4) vs. 31 (IC_50_ = 18.19 nM, com_lipohyd_3A = 1), to list a few.

As a consequence, for the first time, com has been used to explain the difference in the inhibitory activity of several ligands for arginase I. In addition, the innovative approach gives a previously unavailable explanation for discrepancies in the activity of positional isomers.

**FIGURE 6:**
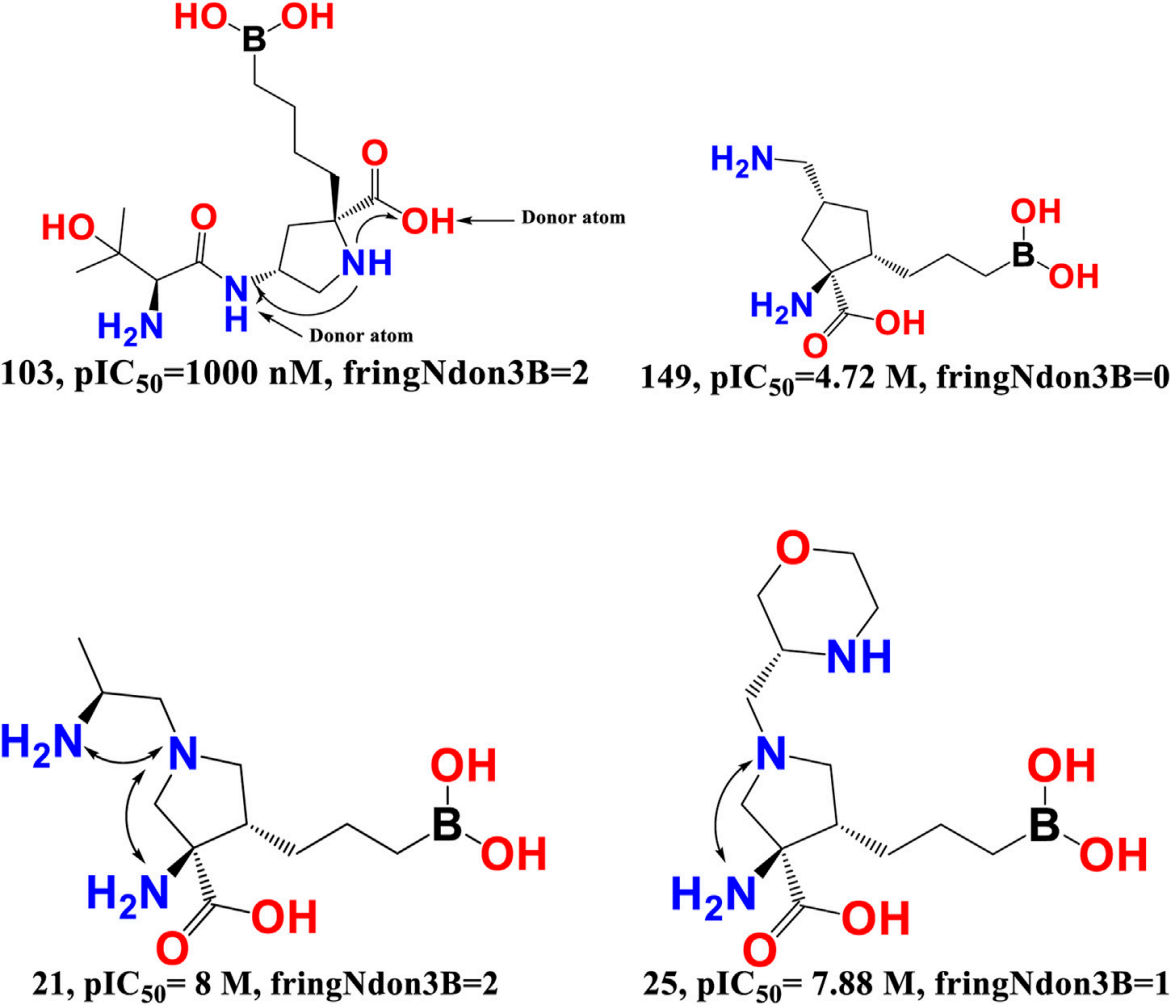
Presentation of the molecular descriptor fringNdon3B for the molecular pairs; 103 and 149, and for 21 and 25 only.

fringNdon3B- The frequency of occurrence of a donor atom precisely 3 bonds away from the ring nitrogen atom is indicated by the chemical descriptor fringNdon3B. This descriptor offers vital information on the maximum amount of separation needed between the two polar (ring nitrogen atoms and donor atoms) moieties to provide a superior araginase inhibitory activity profile. A ring nitrogen atom makes a positive contribution if it is precisely three bonds away from a donor atom; as a result, this combination must be maintained for a better activity profile. On the other hand, the araginase inhibitory action may be decreased by narrowing the bond gap between the ring nitrogen and donor atom. The obtained results correspond precisely to the pharmacophores (descriptors) described in the QSAR model. Furthermore, the most active molecules had the highest values for the descriptors fringNdon3B and rsa. This shows that increasing the number of donor atoms may also increase the rsa value if the value of fringNdon3B is raised. This observation is well supported by the molecular pair: 103 and 149, 21 and 25 (see Figure 6).

Merck Sharp and Dohme, AstraZeneca, and Sichuan Kelun-Biotech Biopharmaceuticals have independently developed an additional class of arginase inhibitors comprising a proline (pyrrolidine ring) scaffold. They noted that the proline containing an amino group at position 4 (compound 28) was positioned precisely three bonds from the donor hydroxy segment of the carboxyl moiety and had an IC_50_ value of 3.2 nM for human ARG-1 in the TOGA experiment (Mitcheltree et al., 2020). This observation highlights the importance of the molecular descriptor fringNdon3B, and the QSAR results are perfectly aligned with the reported findings.

**FIGURE 7:**
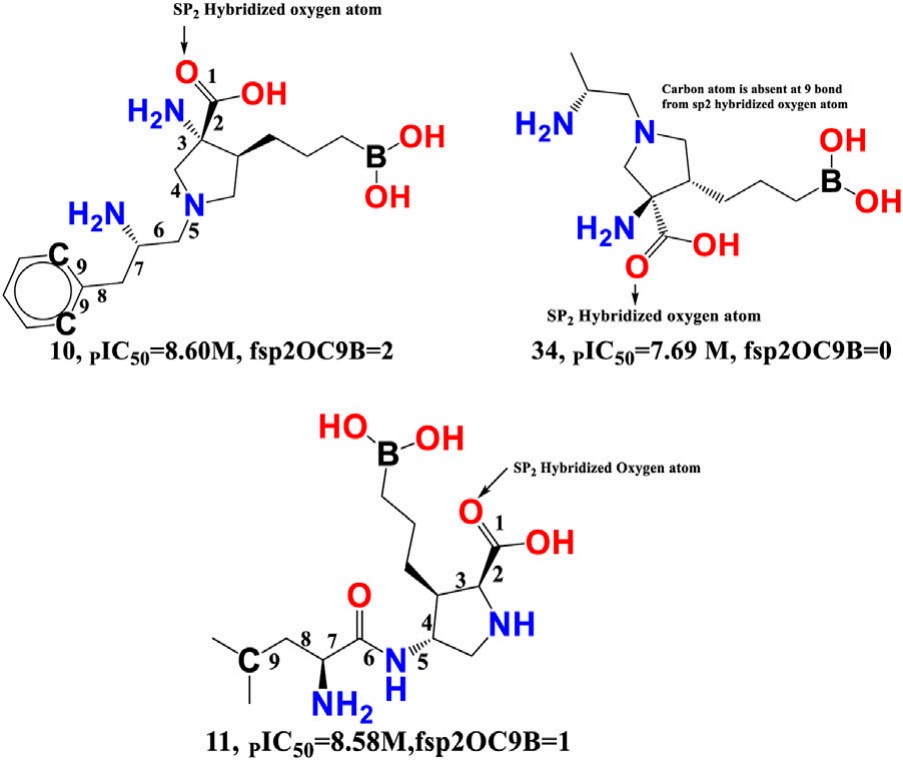
Illustration of the molecular descriptor fsp2OC9B for the molecule 10 (IC_50_ = 2.51 nM, fsp2OC9B = 2), 34 (IC_50_ = 20.4 nM, fsp2OC9B = 0), and 11 (IC_50_ = 2.63 nM, fsp2OC9B = 1) only.

fsp2OC9B-This descriptor refers to the frequency of occurrence of carbon atoms precisely at 9 bonds from the sp2 hybridised oxygen atom, and it has a positive coefficient in the developed QSAR model. If the same carbon atom can be found at 9 bonds from the sp2 hybridised oxygen atom or any other oxygen atom along any route, it was omitted when calculating fsp2OC9B. The importance of fsp2OC9B is demonstrated by the fact that one or more of the most active compounds, such as 10, 11, 21, and 20, with IC50 values ranging from 10.0 to 2.51 nM, contained a carbon and a sp2 hybridised oxygen atom (see Figure 7). With a few exceptions, such as molecule numbers 34, 175, 176, 177, 178, and 179, the reverse is true for less active compounds (IC50 = 7943–870963590 nM).

Van Zandt M. and colleagues have identified a broad family of N-substituted 3-amino-4-(3-boronopropyl) pyrrolidine-3-carboxylic acids as potent third-generation inhibitors of human arginase I and II (Van Zandt et al., 2019). The reported compounds had a sp2-hybridized oxygen atom and precisely nine bonds from the second and sixth carbon atoms of the phenyl ring system, with an IC_50_ of 1.3 nM for the arginase I receptor. Similar pharmacophoric features have been captured in the developed QSAR model as well. Therefore, QSAR results align perfectly with the reported findings.

**FIGURE 8:**
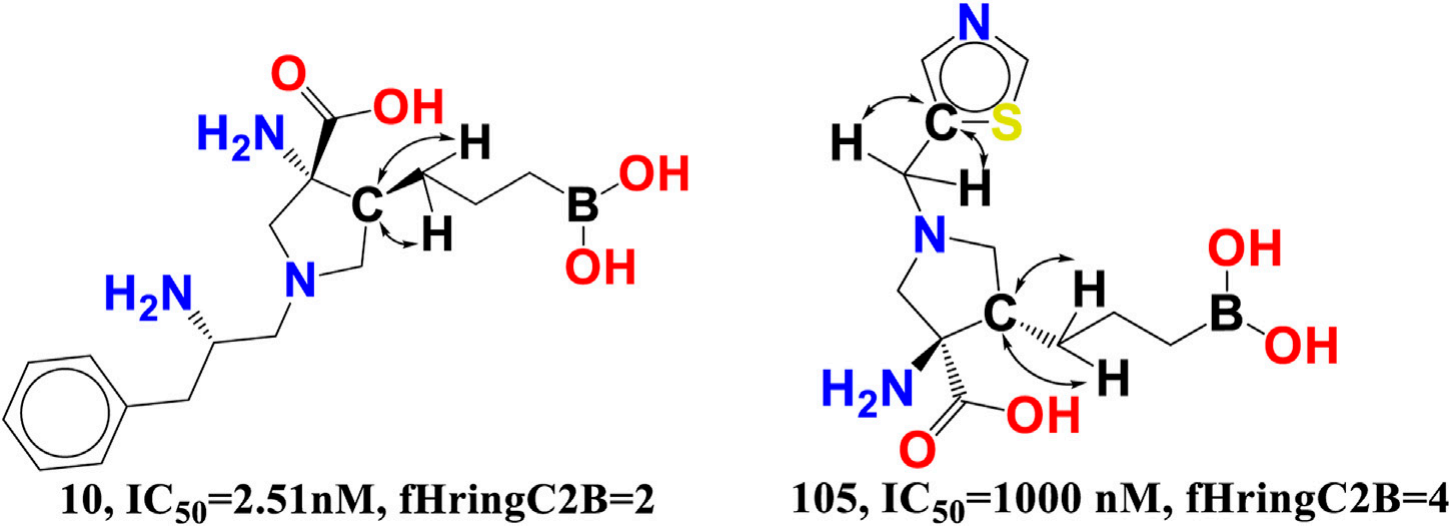
Presentation of the molecular descriptor fHringC2B for the molecules 10 and 105 only.

fHringC2B-This descriptor indicates the frequency of occurrence of ring carbon atoms precisely 2 bonds from the hydrogen atoms. The negative value for fHringC2B suggests that the presence of hydrogen close to the ring carbon reduces the inhibitory action of arginase I. In the majority of reported molecules, fHringC2B exists owing to the direct attachment of hydrogen to the carbon atom (C-H) or due to hydrogen atoms linked to carbon atoms next to the ring carbon atom (C-CHn fragment). This can be observed by comparing the molecules 10 and 105 (see Figure 8).

fHringC2B favours two structural characteristics that might result in an improved arginase inhibitory profile: 1) the presence of hydrophobic hydrogen atoms in C-H or H-C-C fragments 2) steric hindrance or bulkiness in the region of ring carbon atoms due to the fact that hydrogen is the smallest element. Less bulkiness around ring carbon atoms results in a more potent arginase inhibitory profile. These two structural characteristics permit the formation of hydrophobic contacts or arene-cation interactions between the ligand and the receptor.

**FIGURE 9:**
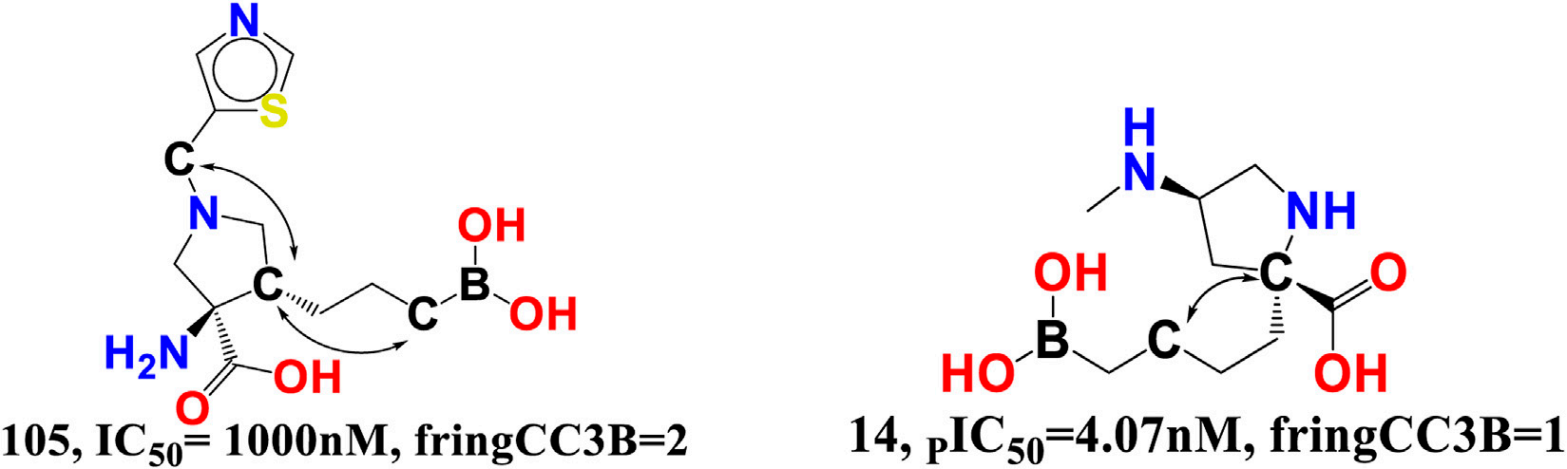
Presentation of the molecular descriptor fringCC3B for the molecules 105 and 14 only.

fringCC3B- The descriptor is related to two characteristics, namely, carbon atoms and ring carbon atoms. As its coefficient in the proposed QSAR model is negative, increasing the amount of these carbon atoms decreased the pIC_50_ value. As these descriptors are also related to carbon, the value of fHringC2B and com_Lipo_hyd 3A might be affected by an increase in fringCC3B. This suggests that pharmacophore synergism determines the ultimate inhibitory ability of a drug towards arginase I receptor. When molecule 14 (IC_50_ = 4.07 nM, fHringC2B = 1, com_Lipo_hyd 3A = 7) is compared to molecule 105 (_P_IC_50_ = 1,000 nM, fHringC2B = 4), this is evident (see Figure 9).

There are 16 compounds in the current dataset that include at least one such carbon and ring carbon combination (fringCC3B). Similarly, 14 of the most active compounds with pIC_50_ values between 8 and 9.09 M, excluding molecules 3 and 9, have fringCC3B > 1. A comparison between molecules 14 and 105 reinforces this conclusion.

### QSAR based virtual screening and drug repositioning

In this investigation, the most modern methodologies, namely, QSAR-based virtual screening and drug repositioning, were combined by estimating the arginase I inhibitory activity (pIC50) of 1,615 clinically approved ZINC FDA compounds. The QSAR VS. predicts the arginase I inhibitory activity (pIC_50_) of all 1615 FDA compounds, of which 112 have a pIC_50_ in the range of 8.07–10.023 M. Therefore, a molecular docking study was conducted to determine the binding pattern of these 112 FDA molecules. Interestingly, one of the topmost predicted FDA molecules (ZINC000252286875) has a docking score of −10,801 kcal/mol and a pIC_50_ of 10.023 M. While other FDA compounds had a higher docking score, they had a relatively low pIC_50_. As a result, the molecule ZINC000252286875 was chosen as a significant repurposed hit in QSAR vs. Table 3 contains the pIC_50_ values for the top 10 FDA compounds, as well as their smiles notations, docking scores, and zinc IDs (See Supplementary Table S4 for the calculated 1,615 molecular descriptors).

**TABLE 3:** Presentation of zinc id, structures, _P_IC_50_ Predicted by QSAR vs, Docking Score (Kcal/mol), and RMSD(Å) for top 10 FDA molecules.

S. No	zinc_id	Smiles notations	_P_IC_50_ by QSAR	Docking score (kcal/mol)	RMSD(Å)
1	ZINC000252286875	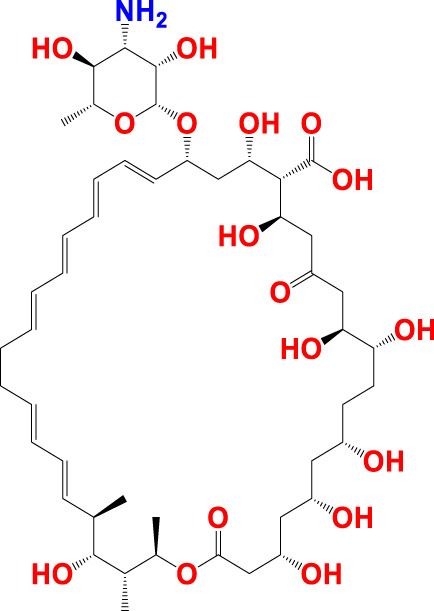	10.023	−10.80	2.130
2	ZINC000252286877	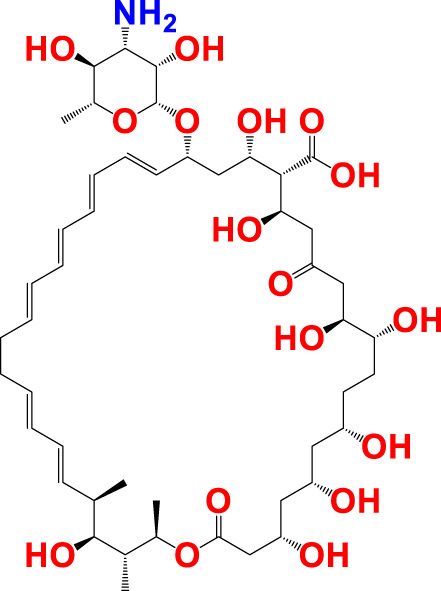	10.156	−10.27	2.58
3	ZINC000169621228	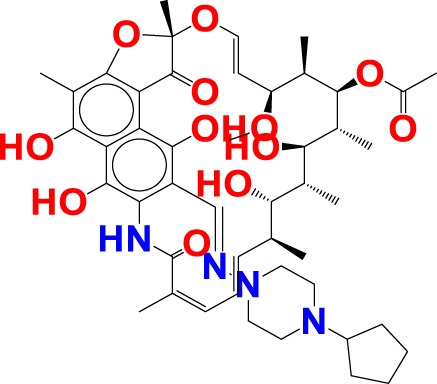	8.048	−9.92	1.74
4	ZINC000218037687	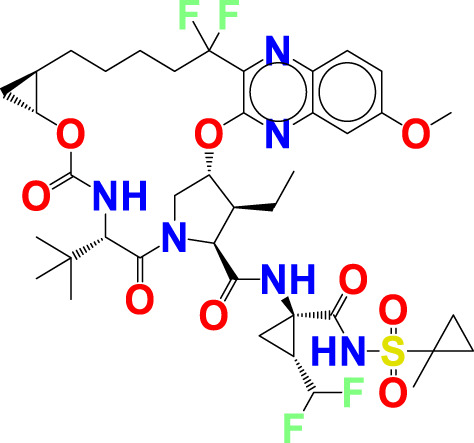	9.89	−9.78	1.74
5	ZINC000203757351	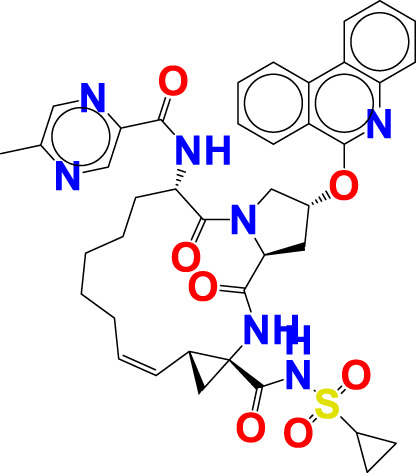	8.643	−9.43	1.66
6	ZINC000095551509	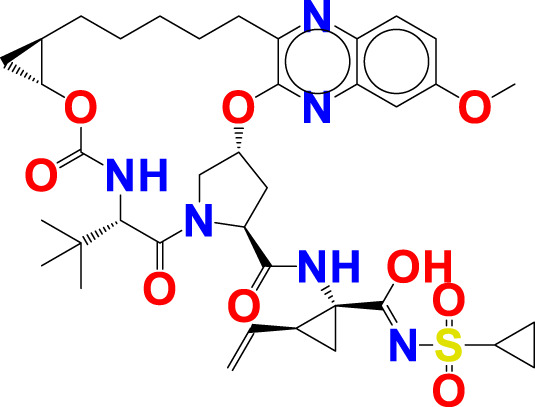	8.351	−9.36	1.53
7	ZINC000252286876	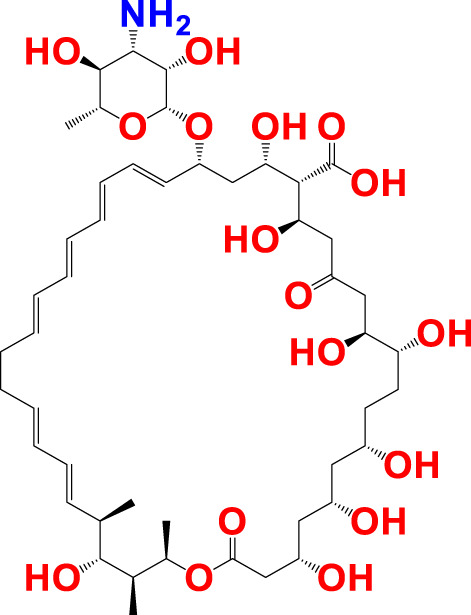	9.937	−9.24	2.21
8	ZINC000150601177	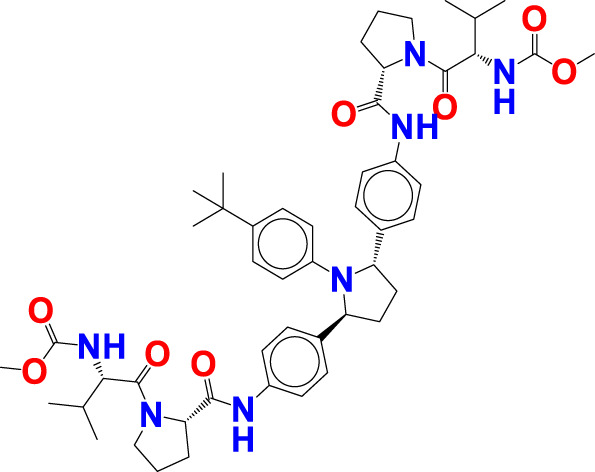	10.709	−9.23	2.09
9	ZINC000028108825	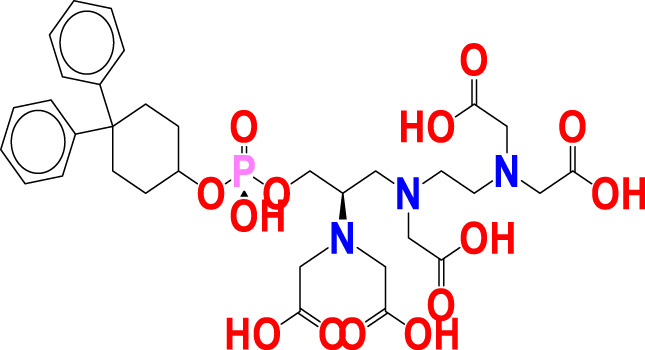	8.473	−9.20	2.60
10	ZINC000085537011	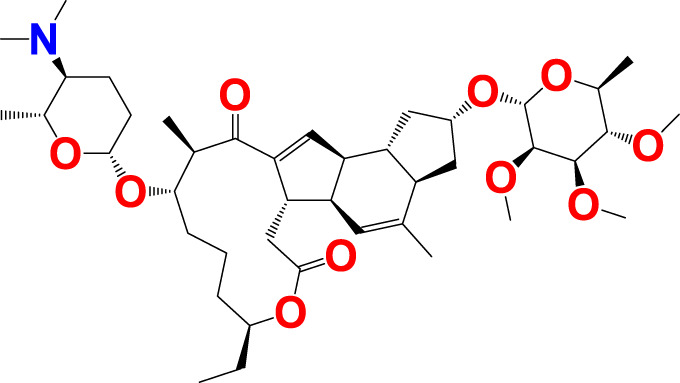	8.172	−8.83	2.12

### Applicability domain analysis of the hit molecules identified in QSAR based VS

**FIGURE 10:**
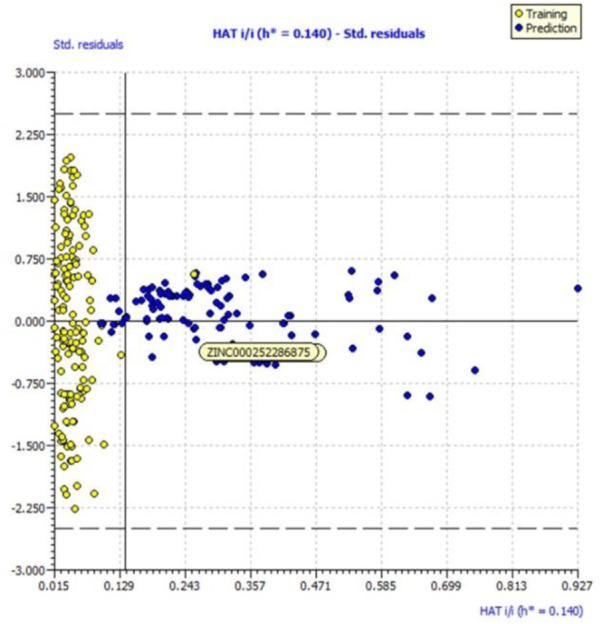
Williams plot for the applicability domain of the top hit molecule (ZINC000252286875) identified in the QSAR based virtual screening (blue dots indicates the hit molecules).

We evaluated the applicability domain of the created QSAR model with respect to the top most active hit molecules found in the QSAR-based virtual screening using a training set of 149 molecules and a prediction set of 112 hit molecules. The top hit molecule, ZINC000252286875, was found in the Williams plot with a low leverage value of HAT i/i h* = 0.140, which is on the edge of the applicability domain (see Figure 10).

The leverage figures demonstrate how much each chemical’s structure affects the model. The predicted data for the top hit molecule, ZINC000252286875, is therefore acceptable because the low value of leverage suggests that the prediction set is detected as being near the chemical domain of the training set molecules.

### Molecular docking analysis

**FIGURE 11:**
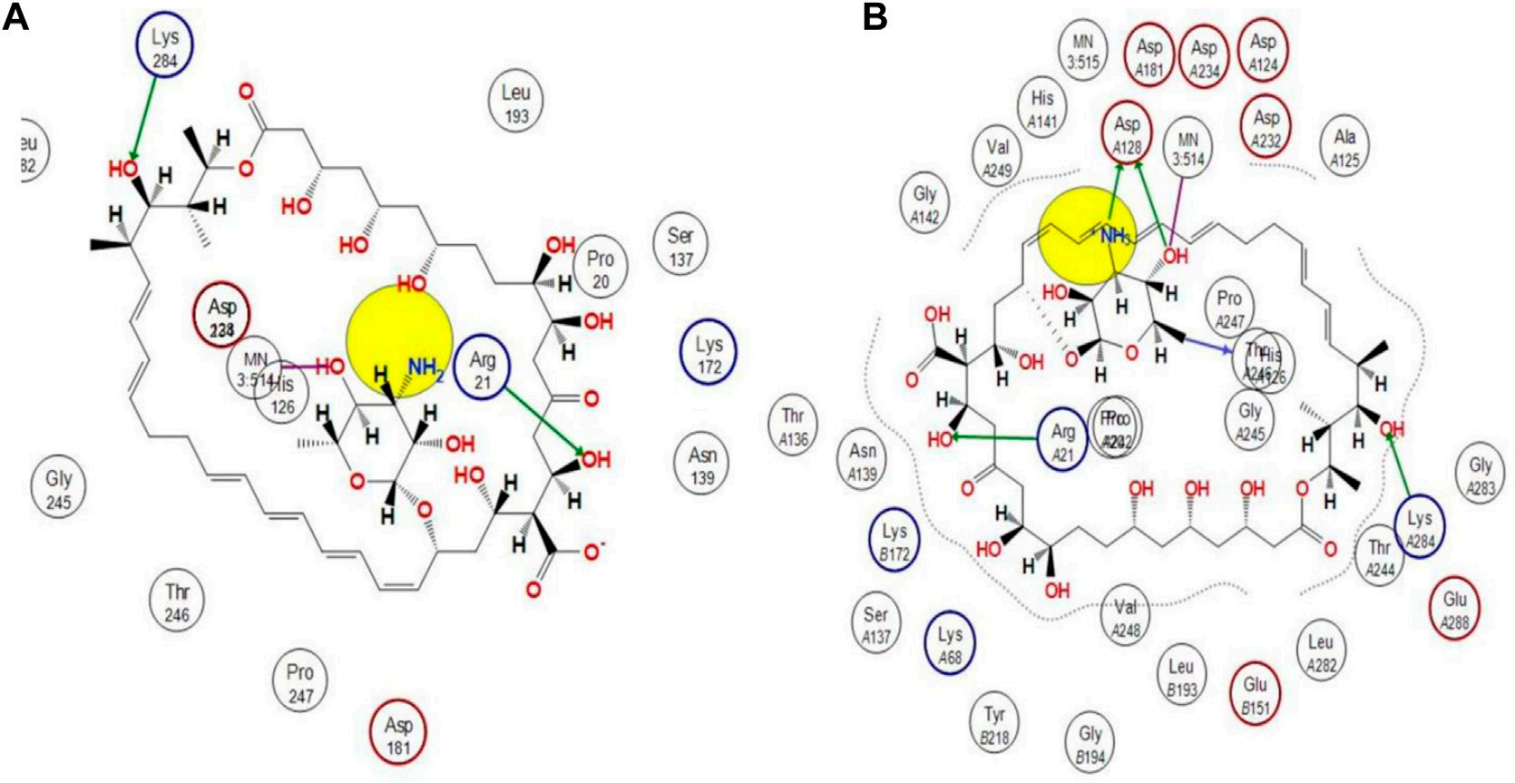
Representation of the 2D interactions of the **(A)** protonated ZINC000252286875 and **(B)** Non-Protonated ZINC000252286875 ligand with the arginase-1 protein (pdb-3KV2).

Using docking simulations on hARG I, the binding modes of the most active hit molecule, ZINC000252286875, were identified in order to learn more about the inhibitory mechanism (pdb id: 3kv2). Although b-ARG I’s structure is not included in the Protein Data Bank, the active site of the two molecules is 100% identical. Docking simulations were used to identify the binding modes of the identified hit molecule ZINC000252286875 in the QSAR-based VS. (pdb id: 3kv2) to gain more insight into the inhibitory mechanism. Using an NRG Suite docking software accessible as a PyMOL plugin, the top active hit molecule, ZINC000252286875, and the pdb-3kv2 ligands were docked successfully into the active pocket of h-ARG I. Table 3 shows the structures, docking score (kcal/mol), RMSD, and predicted _p_IC_50_ values from QSAR-based virtual screening. We chose the most active molecule based on the highest docking score as well as the highest pIC_50_ value. As a result, we picked the compound ZINC000252286875 for the drug receptor interaction study. The docking findings revealed that the ZINC000252286875 molecule had polar and non-polar interactions with arginase I. The docking score was −10.8010 kcal/mol (RMSD = 2.13) (see Figure 11). Intriguingly, ZINC000252286875 entered the protonation state during drug-receptor interactions. It formed hydrogen bonds with arginase I in both protomeric forms, but the number of H-bonds varied. In its natural state, a molecule exhibits hydrogen bonding connections with Lys284 and Arg21 through its hydroxy group and forms a coordination complex with the MN3:514 metal. Furthermore, in its natural state, the molecule induced ionisation of the O-H moiety, resulting in metal-bridging oxyanion. Interestingly, L. Di Costanzo et al. noted the potential of the same finding (Di Costanzo et al., 2010). However, hydrogen atom positions cannot be determined by x-ray crystallography, hence theoretical predictions of protonation states are necessary (ten Brink and Exner, 2009).

The protonation state of the molecule is associated with the fluctuation in the _P_H in the receptor binding pocket. Particularly, each amino acid’s functional group becomes protonated at a pH value below its pKa. A functional group is deprotonated when the pH rises over its pKa. When the pH is equal to the pKa, the functional group is neutral, with equal numbers of protons and negative ions. The ASP128 residue is only connected to the molecule ZINC000252286875 *via* interactions with the amine and OH moiety on the pyran ring (see Figure 11B). Based on this finding, it is possible that proton uptake is the key regulatory switch for modulating the function of the arginase 1 receptor. In the protonation state, the NH_2_ moiety underwent protonation, resulting in the formation of NH_3_^+^. Based on this observation, the molecule ZINC000252286875 displayed more firm binding in its protonated state than in its native form. The increased frequency of H-bond interactions reveals this. In its protonated state, the hydroxy group of the molecule ZINC000252286875 formed two hydrogen bonds with Arg21 and Lys284, while another hydroxy group and protonated amino moiety (NH3+) formed two more hydrogen bonding connections with Asp128. In addition to this, it formed an additional hydrogen bond with the residue Thr246. Moreover, the protonated version of the molecule formed a metal coordination complex with MN3514 through a 2-hydroxy group on the pyran ring. The effects of molecule pKi and protonation state on the ligand’s affinity for the receptor are shown by Alexey V. Onufriev and colleagues (Onufriev and Alexov, 2013). They further went on to explain that the receptor-ligand binding becomes pH-dependent when the protonation state changes at a certain pH. Subsequently, the researchers claimed that the drug receptor interactions in the protonated state result in a structural reorganisation (change in conformation). This was discovered to be true in the case of molecule ZINC000252286875 in terms of protonation and non-protonation states. Further, a literature survey revealed that these modifications (structural reorganization) are the direct result of binding-induced alterations in the pKi values of ionizable groups in proteins and their respective ligands (Onufriev and Alexov, 2013). Therefore, the reported observation was supporting the protonation state acquired by the molecule ZINC000252286875 during the drug receptor interactions. Further, the protonation state of the molecule ZINC000252286875 is only linked to the alteration in the pKi value of the ASP128 residue through interactions with the amine and OH groups on the pyran ring. Furthermore, this observation revealed that the structure reorganization (change in conformation) in protonated and non-protonated states was linked with the fluctuation in pki values of the amino acid residues in arginase-1 protein. This observation was supported by the results reported by Alexey V. Onufriev and colleagues (Onufriev and Alexov, 2013).

### Molecular dynamics simulation (MD) analysis

Molecular dynamics and simulation (MD) studies were done to figure out the stability and convergence of the ZINC000252286875-bound Arginase-1 (PDB ID: 3KV2) complex. Root mean square deviation (RMSD) values showed that the conformation at each 500-ns simulation was stable. The root mean square deviation (RMSD) is a way to figure out how much a group of atoms moves away from a reference frame on average. It is worked out for every single frame of the trajectory. The RMSD for frame x is:

$$RMSD_{x}=\sqrt{\frac{1}{N}\sum_{i=1}^{N}{\left(r_{i}^{\prime }\left(t_{x}\right)-r_{i}\left(t_{ref}\right)\right)}^{2}}$$

Where N is the number of atoms in the atom selection; t_ref_ is the reference time (the first frame is usually used as the reference and is treated as time t = 0); and where r' is the position of the selected atoms after superimposing on the reference frame in frame x, where frame x is recorded at time t_x_. Every frame in the simulation trajectory is subjected to the same technique (Brown et al., 1996). For characterising local changes along the protein chain, the Root Mean Square Fluctuation (RMSF) is useful. The RMSF for residue I is:

$$RMSF_{i}=\sqrt{\frac{1}{T}\sum_{i=1}^{T}\langle {\left(r_{i}^{\prime }\left(t\right)-r_{i}\left(t_{ref}\right)\right)}^{2}\rangle }$$

The angle brackets indicate that the square distance is averaged over the residue’s atom selection. Where T is the trajectory time used to calculate the RMSF, tref is the reference time, and ri is the position of the residue. The position of atoms in residue I after superposition on the reference is given by r′. Desmond’s simulation paths were investigated. The root mean square deviation (RMSD), root mean square fluctuation (RMSF), and protein-ligand interactions were calculated using MD trajectory analysis. Protein RMSD evolution: The graphs show the evolution of a protein’s RMSD (left *Y*-axis). Once all protein frames are aligned on the reference frame backbone, the RMSD is calculated based on atom selection.

**FIGURE 12:**
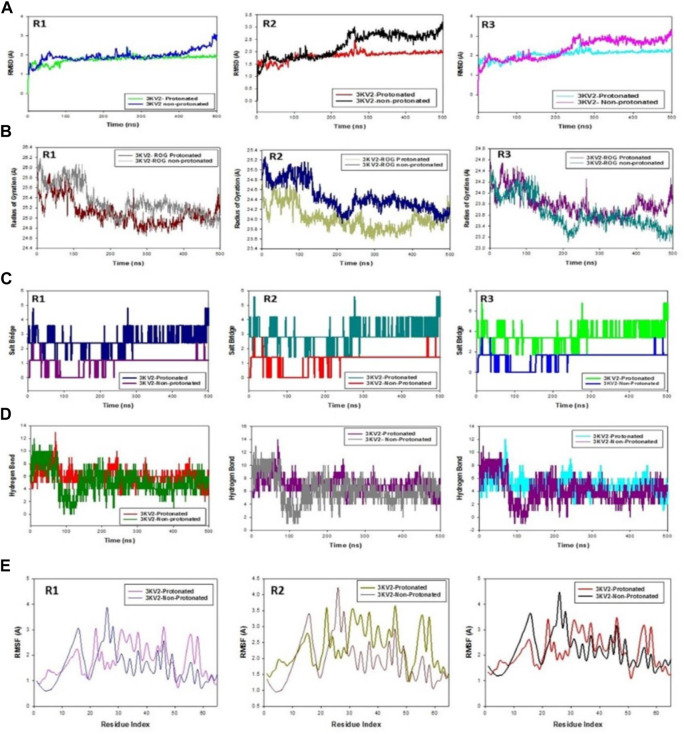
**(A)** MD simulation trajectory analysis of RMSD of ZINC000252286875 bound with 3KV2, i.e., Arginase-1,500 ns time frame in triplicate displayed: R1 (replicate 1) RMSD plot of ZINC000252286875 bound Arginase-1 (PDB I.D: 3KV2) in Protonated (green) versus non-Protonated protein Arginase-1 (PDB I.D: 3KV2) (blue); R2 (replicate 2) RMSD plot of ZINC000252286875 bound Arginase-1 (PDB I.D: 3KV2) R1 (replicate 1) ROG plot of ZINC000252286875 bound Arginase-1 (PDB I.D.: 3KV2) in protonated state (light grey) versus non-protonated bound protein Arginase-1 (PDB I.D.: 3KV2) (chocolate brown); R2 (replicate 2) Arginase-1 (PDB I.D.: 3KV2) in protonated state (navy blue) with non-protonated bound Arginase-1 (PDB I.D.: 3KV2) (light olive); R3 (replicate 3) ROG plot of ZINC000252286875 bound Arginase-1 (PDB I.D.: 3KV2) in protonated state (pink) with non-protonated bound protein C. MD simulation trajectory analysis of Salt Bridge of ZINC000252286875 bound with Arginase-1 in protonated state (PDB I.D.: 3KV2) with non-protonated state at 500 ns time frame in triplicate displayed: R1 (replicate 1) ZINC000252286875s Salt Bride plot is bound. Arginase-1 (PDB I.D.: 3KV2) in protonated state (PDB I.D.: 3KV2) (navy blue) with non-protonated bound protein R2 (replicate 2) Salt bridge plot of ZINC000252286875 bound Arginase-1 (PDB I.D.: 3KV2) in protonated state (teal) versus non-protonated bound state with protein Arginase-1 (PDB I.D.: 3KV2) (red); R3 (replicate 3) Salt Bridge plot of ZINC000252286875 bound Arginase-1 (PDB I.D.: 2ZHV) in protonated state (lime green) with protein Arginase-1 (PDB I.D.: 3KV2) (blue); **(D)** MD simulation trajectory analysis of Arginase-1 (PDB I.D. R1 (replicate 1) R2 (replicate 2) H-bond plot of ZINC000252286875 bound protonated ligand (red) and non-protonated ligand (green) with Arginase-1 (PDB I.D.: 3KV2) (red). R3 (replicate 3) H-bond plot of ZINC000252286875 bound protonated ligand (dark grey) and non-protonated ligand (magenta) with Arginase-1 (PDB I.D.: 3KV2). ZINC000252286875 H-bond plot with Arginase-1 bound protonated ligand (cyan) and non-protonated ligand (magenta) (PDB I.D.: 3KV2). R1 (replicate 1) RMSF plot of ZINC000252286875 bound Arginase-1 (PDB I.D: 3KV2) in protonated form (magenta) and non-protonated bound form with protein Arginase-1 (PDB I.D: 3KV2) (blue); R2 (replicate 2) RMSF plot of ZINC000252286875 bound Arginase-1 (PDB I.D: 3KV2).

The C-backbone of arginase-1 bound to protonated ZINC000252286875 exhibited a deviation of 2.9 (Figure 12A R1, R2, and R3), while non-protonated ZINC000252286875 displayed a RMSD of 1.8. RMSD plots are within the acceptable range, signifying the stability of proteins in the protonated as well as non-protonated ZINC000252286875 bound state before and after simulation, and it can also be suggested that the fact that the non-protonated ZINC000252286875 bound arginase-1 (PDB I.D.: 3KV2) is quite stable in complex might be due to significant binding of the ligand. Additionally, the radius of gyration is a measure of the compactness of the protein. Proteins had a Rg value of 25 in the protonated-ZINC000252286875 bound state, indicating a decrease in the radius of gyration (Rg) (Figure 12B; R1, R2, and R3), whereas non-protonated-ZINC000252286875 had a Rg of 25.2, indicating the compactness of the protein-ligand complex in both the protonated and non-protonated states. From the overall quality analysis from RMSD and Rg, it can be suggested that protonated as well as non-protonated ZINC000252286875 bound to the protein targets posthumously in the binding cavities and played a significant role in the stability of the proteins.

Significant root mean square fluctuations (RMSF) were seen in the arginase-1 protein at a small number of residues for a time function of 500 ns in both the protonated and unprotonated states. On the RMSF plot, peaks represented regions of the protein that experienced the most change throughout the course of the simulation. Protein tails (both N- and C-terminal) are the most dynamic regions of the molecule. Secondary protein structures like alpha helices and beta strands are often more stable than loop regions and less flexible than the unstructured protein core.

Based on MD trajectories, we know that the residues with the highest peaks are located in loop regions or the N- and C-terminal zones. (Figure 12E, R1, R2, and R3). Low RMSF values of binding site residues are indicative of stable ligand binding to the protein. Figure 6 shows results obtained from three distinct arginase-1 studies. Figure 6 shows that the complex is stabilized, despite the presence of a few fluctuating peaks. In this case, the RMSF values are suitable for stabilising the protein-ligand complex. Protein structures were shown to fluctuate more in the non-protonated state than in the protonated state during simulation in the ZINC000252286875-bound conformation, as demonstrated by RMSF plots.

**FIGURE 13:**
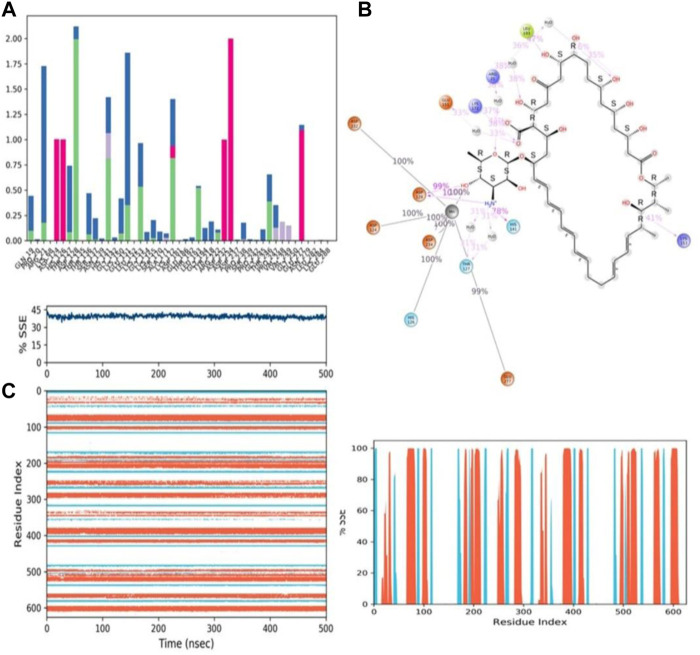
**(A)** Protein-ligand contact histogram (H-bonds, Hydrophobic, Ionic, Water bridges) of the PROTONATED STATE OF THE ligand, ZINC000252286875 bound with 3KV2 recorded in a 500 ns simulation interval; **(B)** Ligand atom interactions with the protein residues of 3KV2 bound with ZINC000252286875; **(C)** Secondary Structure element distribution by residue index throughout the protein structure. Red indicates alpha helices, and blue indicate beta-strands of 3KV2 bound with protonated ZINC000252286875.

During the 500-ns simulation, the average number of hydrogen bonds and salt bridge contacts established between ZINC000252286875 and the corresponding protein, arginase-1 (PDB I.D.: 3kv2), were also recorded (Figures 12C, D). Tri-replicate MD simulations of ZINC000252286875 with arginase-1 showed hydrogen bond formation from 0 to 500 ns (Figure 12D; R1, R2, and R3). Following 500 ns of molecular dynamics, the number of hydrogen plots analysed confirmed the docking pattern of two hydrogen bond formation with arginase-1 (PDB I.D.: 3kv2) (Figure 12D). While in simulation, the binding of arginase-1 to ZINC000252286875 was reinforced by the number of hydrogen bonds and salt bridges (Figure 12C; R1, R2, and R3) formed between the two molecules. The “Simulation Interactions Diagram” tab in Maestro shows the various subtypes of each interaction type (see Figure 13A). The stacked bar charts have a consistent appearance throughout the trajectory. Because the same type of interaction can occur multiple times between the ligand and the same protein residue, values greater than 1.0 are possible.

**FIGURE 14:**
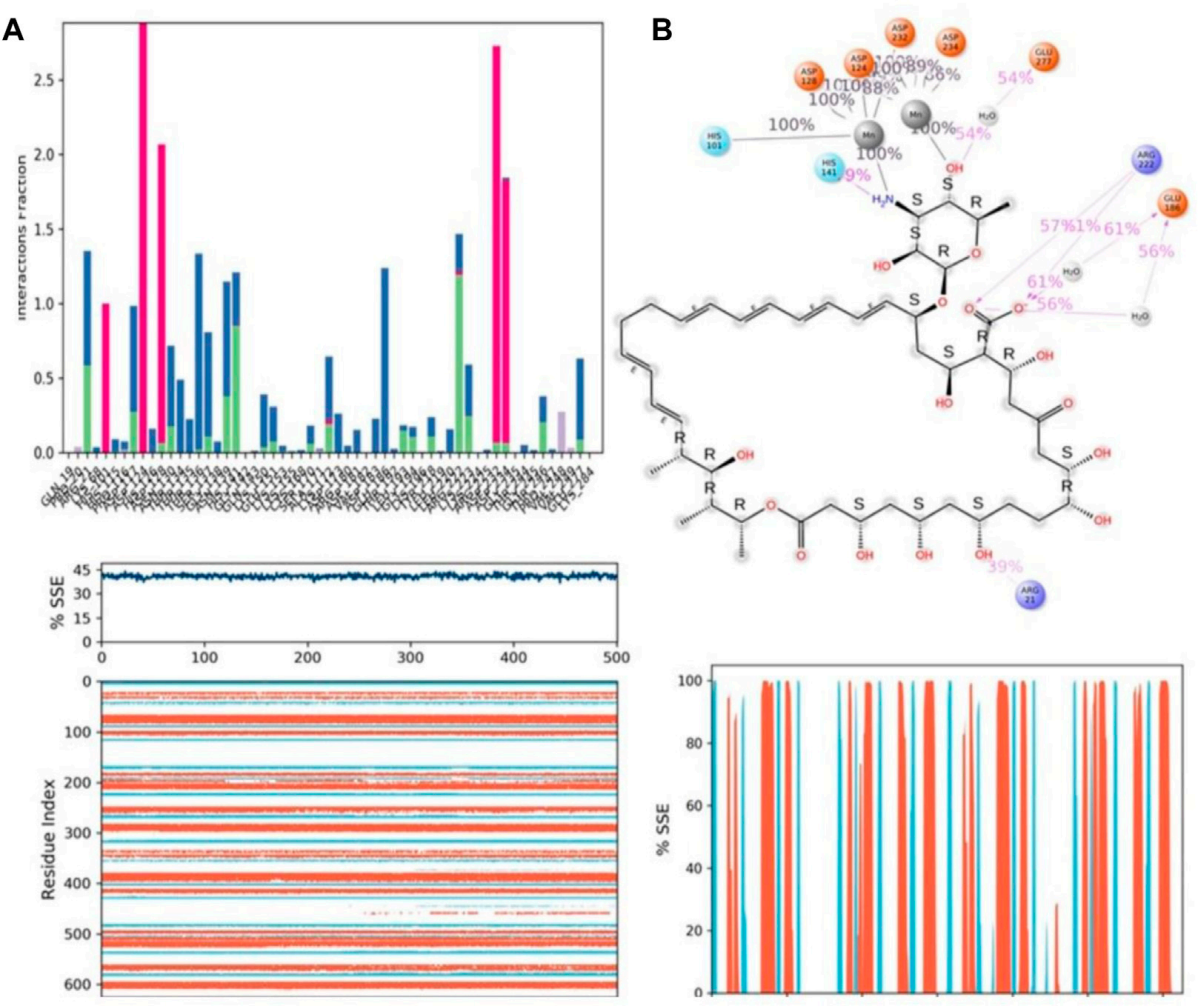
**(A)** Protein-ligand contact histogram (H-bonds, Hydrophobic, Ionic, Water bridges) of the non-protonated state of the ligand, ZINC000252286875 bound with 3KV2 recorded in a 500 ns simulation interval; **(B)** Ligand atom interactions with the protein residues of 3KV2 bound with ZINC000252286875; **(C)** Secondary Structure element distribution by residue index throughout the protein structure. Red indicates alpha helices, and blue indicate beta-strands of 3KV2 bound with non-protonated ZINC000252286875.

Protein–ligand interactions were seen throughout the simulation for both protonated and non-protonated ligands. These interactions can be grouped and summarised according to the types shown in the preceding graph. Protein-ligand interactions (or “contacts”) come in four different types: hydrogen bonds, hydrophobic interactions, ionic interactions, and water bridges. In its protonated form, the ligand ZINC000252286875 exhibited six ionic interactions with Lys64, Asp124, Ala171, Arg222, Asp232, and Gly250. Aspartic acid residue 232 exhibited a 200% ionic contact, while other residues exhibited ionic contacts above 100%. Which suggests that ionic connections were maintained during the 500 ns simulation time (see Figure 13B). In the docking study, salt bridge formation for the ligand ZINC000252286875 was observed, thereby complementing the docking analysis. The ligand ZINC000252286875 made six ionic contacts with the Lys68, Asp117, His126, Ala171, Lys224, and Asp232 residues in a non-protonated state; Asp117, His126, and Lys224 made more than 200% ionic contacts, while the remaining residues made more than 100% ionic contacts (See Figure 14B). The ligand ZINC000252286875 formed ionic interactions with distinct residues in both protonated and non-protonated states, with the exception of Asp232 and Ala171, which formed ionic contacts in both protonated and non-protonated states. In the protonated state, the ligand ZINC000252286875 formed strong ionic connections with Asp232, but in the unprotonated state, it formed strong ionic interactions with Asp117, His126, and Lys224. The current observation demonstrated that the ligand ZINC000252286875 exhibited more stable interactions in its non-protonated state than in its protonated state. The ligand ZINC000252286875 subsequently exhibited significant hydrogen bonding interactions in both the protonated and unprotonated states. When the ligand was protonated, it made a strong hydrogen bonding contact of 200% with Thr127. Other residues, such as Prp20, His126, Thr135, Asn139, Gly132, Lys150, Leu152, Gly154, Thr192, Tyr218 and Gly245, also made strong hydrogen bonds during a 500 ns simulation. In contrast, the non-protonated form of ligand ZINC000252286875 had only one significant hydrogen bonding interaction with the Leu220 residue (11%), but it had many hydrogen bonding contacts with the Pro20, Pro116, His126, Asp128, Gly138, Asn139, Gln143, Lys150, Cys168, Ala171, Thr 192, Leu192, Lys196, Arg222, etc. Although the ligand demonstrated hydrogen bonding interactions in both forms, it displayed more stable hydrogen bonding connections in its protonated form Figure 14C. Throughout the simulation, the existence of protein secondary structural elements (SSE) such as alpha heices and beta strands is examined to ensure that they are not present. The plot in Figure 14C depicts the distribution of SSE by residue index over the complete protein structure, and it encompasses the full protein structure. In contrast to the charts, which show the summary of the SSE composition for each trajectory frame during the course of the simulation, the graphs at the bottom show the evolution of each residue and its SSE assignment throughout the experiment. Throughout the simulation, alpha-helices and beta-strands are monitored as secondary structure elements (SSE). The left graph shows the distribution of SSE across the protein structure by residue index. The top image highlights the SSE composition for each trajectory frame throughout the simulation, while the bottom plot tracks each residue’s SSE assignment through time.

Figure 7(iv) is a ligand torsion map showing the conformational changes that occur to each rotatable bond (RB) in the ligand as the simulation progresses (from 0.00 to 500 ns). An abstract, two-dimensional representation of a ligand with rotatable bonds indicated by color is displayed in the upper panel. For each bond torsion that can be rotated, both a dial plot and a bar plot of the same color are provided.

Dial (or radial) graphs show how the torsion’s conformation changed over the course of the simulation. From the origin of the simulation at the centre of the radial plot, the time progression of the simulation is shown radiating outward. The bar plots are a summary of the data from the dial plots. They show the probability density of torsion in the data. If torsional potential data is also available, the graph will also show the potential of a rotatable bond (by summing the potential of the related torsions) in kcal/mol. On the left side of the graph, the *Y*-axis is marked with the potential values in kcal/mol. The correlations between the histogram and the torsion potential can show the conformational strain that the ligand is under to stay in a state where it is bound to a protein (See Supplementary Figure S5A).

A stepwise trajectory analysis revealed the positional change relative to the initial 0 ns structure after 500 ns of simulation time with arginase-1 in ZINC000252286875 protonated and non-protonated states (See Supplementary Figure S5B). ZINC000252286875 has been shown to possess structural angular movement at the end frame to achieve its conformational stability and convergence.

### Molecular mechanics generalized born and surface area (MMGBSA) calculations

The MMGBSA method is useful for comparing the binding energies of ligands to protein molecules in their protonated and non-protonated states. Non-bonded interaction energies were also taken into account when estimating the binding free energy of the various arginase-1-ZINC000252286875 complexes. The binding energy of ZINC000252286875 with arginase-1 was measured to be −43.789 kcal/mol when the ligand was not protonated, and −19.058 kcal/mol when it was. Total binding energy is different for a ligand in its protonated versus non-protonated state in a drug receptor complex. When a molecule is deprotonated, Gbind is ruled by interactions that are not bonds, such as G_bind_Coulomb, G_bind_Covalent, G_bind_Hbond, G_bind_Lipo, G_bind_SolvGB, and G_bind_vdW. For the both protonated and deprotonated states, the G_bind_vdW, G_bind_Lipo, and G_bind_Coulomb energies were the most important contributors to the overall binding energy. However, in both the protonated and deprotonated states, the G_bind_SolvGB and G_bind_Covalent energies contributed the least to the overall average binding energies.

Furthermore, arginase-1ZINC000252286875 complexes formed stable hydrogen bonds with amino acid residues, as indicated by high GbindHbond interaction values. G_bind_SolvGB and G_bind_Covalent both made unfavourable energy contributions and were therefore opposed to binding in both protonated and non-protonated states. See Supplementary Figure S5C shows that ZINC000252286875, a ligand for arginase-1, displayed angular momentum in its non-protonated state before and after the simulation (0 ns and 500 ns), whereas its protonated state exhibited a slight angular change in the pose (from curved to straight) between before and after the simulation (See Supplementary Figure S5D). Because of the enhanced binding pocket acquisition and contact with residues, these conformational alterations increase stability and binding energy (see Table 4).

**TABLE 4:** Binding energy calculation of ZINC000252286875 with 3KV2-Protonated and the 3KV2-non-Protonated interaction energies from MMGBSA trajectories.

Energies (kcal/mol)	3KV2-protonated	3KV2-non-protonated
ΔG_bind_	−19.058 ± 8.49	−43.789 ± 23.079
ΔG_bind_Lipo	−14.641 ± 2.308	−12.141 ± 2.480
ΔG_bind_vdW	−63.722 ± 6.597	−64.1591 ± 7.2456
ΔG_bind_Coulomb	−10.233 ± 9.197	−8.0198 ± 17.84
ΔG_bind_H_bond_	−4.829 ± 1.051	−5.8410 ± 1.4607
ΔG_bind_SolvGB	68.272 ± 8.097	41.551 ± 17.909
ΔG_bind_Covalent	6.095 ± 3.794	4.8208 ± 3.829

**FIGURE 15:**
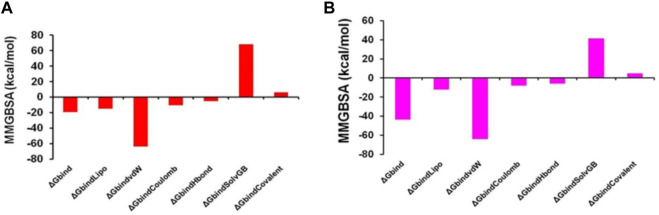
Graphical depiction of Binding energies of ZINC000252286875 for **(A)** 3KV2-Protonated and the **(B)** 3KV2-non-Protonated interaction energies from MMGBSA trajectories.

Thus, MM-GBSA calculations resulted from MD simulation trajectories and were well justified by the binding energy obtained from docking results (See Figure 15 for the graphical depiction of binding energies of protonated and non-protonated complexes). Moreover, the last frame (500 ns) of MMGBSA displayed the positional change of the ZINC000252286875 as compared to the 0 ns trajectory, signifying the better binding pose for best fitting in the binding cavity of the arginase-1 protein (See Supplementary Figure S5). Therefore, it can be suggested that the ZINC000252286875 molecule has good affinity for the major target arginase-1.

In the MMGBSA analysis, we looked at the binding energy contribution of the protonated and unprotonated ZINC000252286875 ligand on a residue-by-residue basis. By calculating the G value of each residue in the 3KV2 complex protonated state, we found that the residue GLN79 contributed the most to the binding free energy in the protonated complex (−65.18 kJ/mol), followed by ASP743, ASN805, and ASN130 (−64.18 kJ/mol, −62.81 kJ/mol, and −59.96 kJ/mol, respectively). In addition, MN832 had the largest contribution to the binding energy among the metals, with a G of −37.02 kJ/mol; MN831, MN514, and MN515 each had lesser contributions, at −34.02 kJ/mol, −17.26 kJ/mol, and −23.5 kJ/mol, respectively. In addition, for the non-protonated ZINC000252286875, the per-residue contribution to the binding energy was also examined. Interestingly, MN 832, with a G of −136.95 kJ/mol, contributed the most to the total binding energy, followed by MN514, −129.78 kJ/mol, MN831, and MN515, each with a G of 126.45 kJ/mol. This finding indicates that the protonated state of the metal-coordinated complex is less stable than the non-protonated form. Moreover, the residue ASN294 with the G of −66.61 kJ/mol contributed the most to the total binding energy, followed by the residues ASN641 (−66.44 kJ/mol), GLN590 (−62.31 kJ/mol), ASN805 (−60.82 kJ/mol), and ASN130 (−60.82 kJ/mol), respectively. We observed that the residue ASN130 is the common contributor in the protonated and non-protonated complexes of the ligand ZINC000252286875. The non-protonated form of the ligand exhibited a more stable binding pattern with the arginase-1 receptor.

### ADMET analysis of the ligand ZINC000252286875

Because of the negative ADMET characteristics, many promising therapeutic medicines fail to enter clinical trials (absorption, distribution, metabolism, elimination and toxicity) (Netzeva et al., 2019; Hassan et al., 2022; Ghufran et al., 2023). The reported chemical ZINC000252286875 (nystatin) is utilised for treatment and prevention in people who are highly immunoexpressed, according to a small number of studies (Gøtzsche and Johansen, 2014). Based on the data shown in Table 5, the following may be concluded: In terms of the percentage of absorption by the human intestines, a number less than 30% indicates a poor absorption rate (Kalantzi et al., 2006). The absorption value of ZINC000252286875 (nystatin) was zero, which assures no absorption by the human gut. In terms of distribution indicators, the size of the distribution (VDss) is said to be large if its value exceeds 0.45 (Pires et al., 2015). The nystatin had a score of −0.215, showing a profile of modest distribution.

**TABLE 5:** Depiction of ADMET results for ZINC000252286875.

Property	Model name	Predicted value	Unit
Absorption	Water solubility	−2.83	Numeric (log mol/L)
Absorption	Caco2 permeability	−1.016	Numeric (log Papp in 10^−6^ cm/s)
Absorption	Intestinal absorption (human)	0	Numeric (% Absorbed)
Absorption	Skin Permeability	−2.735	Numeric (log Kp)
Absorption	P-glycoprotein substrate	Yes	Categorical (Yes/No)
Absorption	P-glycoprotein I inhibitor	No	Categorical (Yes/No)
Absorption	P-glycoprotein II inhibitor	No	Categorical (Yes/No)
Absorption	VDss (human)	−0.215	Numeric (log L/kg)
Distribution	Fraction unbound (human)	0.62	Numeric (Fu)
Distribution	BBB permeability	−2.422	Numeric (log BB)
Distribution	CNS permeability	−6.068	Numeric (log PS)
Metabolism	CYP2D6 substrate	No	Categorical (Yes/No)
Metabolism	CYP3A4 substrate	No	Categorical (Yes/No)
Metabolism	CYP1A2 inhibitior	No	Categorical (Yes/No)
Metabolism	CYP2C19 inhibitior	No	Categorical (Yes/No)
Metabolism	CYP2C9 inhibitior	No	Categorical (Yes/No)
Metabolism	CYP2D6 inhibitior	No	Categorical (Yes/No)
Metabolism	CYP3A4 inhibitior	No	Categorical (Yes/No)
Excretion	Total Clearance	−1.355	Numeric (log mL/min/kg)
Excretion	Renal OCT2 substrate	No	Categorical (Yes/No)
Toxicity	AMES toxicity	No	Categorical (Yes/No)
Toxicity	Max. tolerated dose (human)	−0.028	Numeric (log mg/kg/day)
Toxicity	hERG I inhibitor	No	Categorical (Yes/No)
Toxicity	hERG II inhibitor	No	Categorical (Yes/No)
Toxicity	Oral Rat Acute Toxicity (LD50)	2.421	Numeric (mol/kg)
Toxicity	Oral Rat Chronic Toxicity (LOAEL)	3.195	Numeric (log mg/kg_bw/day)
Toxicity	Hepatotoxicity	No	Categorical (Yes/No)
Toxicity	Skin Sensitisation	No	Categorical (Yes/No)
Toxicity	*T.Pyriformis*toxicity	0.285	Numeric (log ug/L)
Toxicity	Minnow toxicity	9.358	Numeric (log mM)

Blood-brain barrier (BBB) permeability is considered acceptable if the standard value is more than 0.3 and bad if LogBB < −1 (Speciale et al., 2021). In the case of nystatin, BBB penetration is low. For the CNS index, substances with LogPS > −2 are deemed capable of accessing the CNS, while those with LogPS −3 are deemed unable (Han et al., 2019). Nystatin’s distribution indices suggested a greater potential for distribution. In terms of metabolism, cytochrome P450 (CYP) is a crucial detoxifying enzyme. CYP enzymes are found in all bodily tissues (Ouassaf et al., 2021). This enzyme facilitates the excretion of invading germs by oxidising them. Some medications are hindered by cytochrome CYP, while others might be stimulated by it. Inhibitors of this enzyme may alter the medication’s metabolism, and the drug may have the opposite effect (Domínguez-Villa et al., 2021). Hence, it is essential to assess the potential of compounds to inhibit cytochromes (CYP). Up to now, 17 classes of CYPs have been discovered in humans to far. Despite the fact that only CYP1, CYP2, CYP3, and CYP4 are involved in the metabolism of medications, only the kinds (1A2, 2C9, 2C19, 2D6, and 3A4) are responsible for biotransformation for more than 90% of pharmaceutical drugs passing the first phase of metabolism (Zanger and Schwab, 2013). The enzymes 2D6 and 3A4 are the primary drug-metabolizing enzymes (Rodrigues-Junior et al., 2020). Research indicates that nystatin does not inhibit the activity of the aforementioned enzymes. To estimate the metabolic effect of CYP3A4 on the activity of the nystatin that is suggested to use as a medicine, we are dependent on the findings of a research examining the activity of nystatin on the CYP3A4 enzyme (inhibitor or substrate). The data on nystatin’s metabolic properties show that it does not function as a substrate for CYP3A4 and does not block its activity, either.

This shows that nystatin is well tolerated in terms of its metabolism as a medication, and that it reaches its therapeutic target before being oxidised and eliminated. In order to maintain steady medication concentrations, appropriate dose must be based on the drug’s clearance index and excretion characteristics in which the kidneys excrete and the liver clears (Pires et al., 2015). Hence, a higher clearance index value implies that nystatin is eliminated more slowly from the body. In this work, we assess the excretion property of nystatin to estimate the drug’s stability in the body prior to excretion. Based on the index’s predictive values, we know that molecule 16 has a total clearance index of −1.355, suggesting that nystatin may remain in the body for an extended period of time. This increased stability of nystatin in the body over time likely explains why a lower dose of the drug was able to inhibit the enzyme arginase-1 with the same degree of effectiveness. Toxicities of the anticipated substances need to be checked as part of the toxicity indicator. While choosing medications, the letter indication is crucial. The AMES test is often used to determine whether or not a substance is hazardous (Ferraz et al., 2011). Consequently, in this study, we use the AMES test to make inferences about the toxicity of the compounds (nystatin). The investigation demonstrated that nystatin has no toxicity (Pires et al., 2015). We conclude that nystatin satisfies all of the studied pharmacokinetic requirements based on the findings of our *in silico* ADMET characteristics investigation. Thus, nystatin has potential as a drug for inhibiting arginase-1’s enzymatic activity in immunotherapy.

## Conclusion

There is increasing interest in finding novel arginase-I inhibitors due to its crucial involvement in the depletion of arginine, which in turn reduced the proliferation of T and NK cells and eventually resulted in the escape of the immune response to the tumor. However, only a small number of them are now being further investigated, likely as a result of the difficulty in achieving an acceptable pharmacokinetic profile and probable target-related toxicity. Despite these challenges, two arginase inhibitors are being tested in clinical trials: CB-280 for the treatment of cystic fibrosis and numidargistat for the treatment of cancer (phases 1/2 and 1). AstraZeneca’s compound and OATD-02 are two other compounds that are either in preclinical or development stages. Therefore, to achieve this, a QSAR model was used to assess 149 arginase-I inhibitors that have previously been described in the experimental literature. In relation to the various factors that influence the inhibitory activity of arginase-I, the current QSAR analysis has successfully highlighted the significance of various atom kinds, groups, and patterns. Specifically, the rsa descriptor highlighted the relevance of the single carbon atom, which demonstrated a significant alteration in the bioactivity profile of the molecule. More lipophilic (non-polar hydrogen) atoms were shown to increase arginase-I inhibitory activity, as determined by the descriptor com_lipohyd_3A, which strongly suggests including lipophilic (non-polar hydrogen) atoms in future drug design. The importance of precise patterns of atoms with varied levels of hybridization and their interactions in defining the ultimate activity was also highlighted. Moreover, QSAR was also successful in detecting the conditional presence of a sp2-hybridized oxygen atom, precisely nine bonds from the second and sixth carbon atoms of the phenyl ring system, which was also extensively documented in literature. The developed QSAR model possesses high external predictive ability and robustness for fitting and internal validation. The current study used QSAR-based virtual screening to find a unique hit, ZINC000252286875 (Nystatin, _P_IC_50_: 10.023 M, IC_50_: 0.095 nM), as a repurposed molecule for targeting the arginase-I receptor at nanomolar concentrations. In addition, virtual screening successfully offered a potent hit molecule, nystatin, from the ZINC FDA database with improved IC_50_ values in the range of 0.8 to 0.095 nM. The protonated state of the ligand ZINC000252286875 was discovered in the binding pocket of arginase-I by molecular docking investigations. Molecular docking and MD studies revealed polar and non-polar interactions with key arginase-I active site residues. Furthermore, the much greater binding energy of ZINC000252286875 with arginase-I validates the increased affinity and opens up a new avenue for potential arginase-I inhibitor drugs. The non-protonated ZINC000252286875 revealed that the MN 832 contributed the most to total binding energy, with a G of −136.95 kJ/mol, followed by MN514, -129.78 kJ/mol, MN831, and MN515, each with a G of 126.45 kJ/mol. The ligand’s non-protonated form had a more stable binding pattern with the arginase-1 receptor. On the basis of the results of the *in silico* ADMET characteristics analysis, nystatin likewise meets all of the investigated pharmacokinetic criteria. Therefore, the current findings could help in the development of a novel drug for arginase-I inhibition as an onco-immunomodulator.

## Data Availability

The original contributions presented in the study are included in the article/Supplementary Material, further inquiries can be directed to the corresponding authors.
