# Supplementary material for: In-silico studies to recognize repurposing therapeutics toward arginase-I inhibitors as a potential onco-immunomodulators
Source: Front Pharmacol. 2023 Apr 18;14:1129997. doi: 10.3389/fphar.2023.1129997 (PMC10151555; doi:10.3389/fphar.2023.1129997)
Supplement: Supplementary file 3 [file DataSheet5.docx]

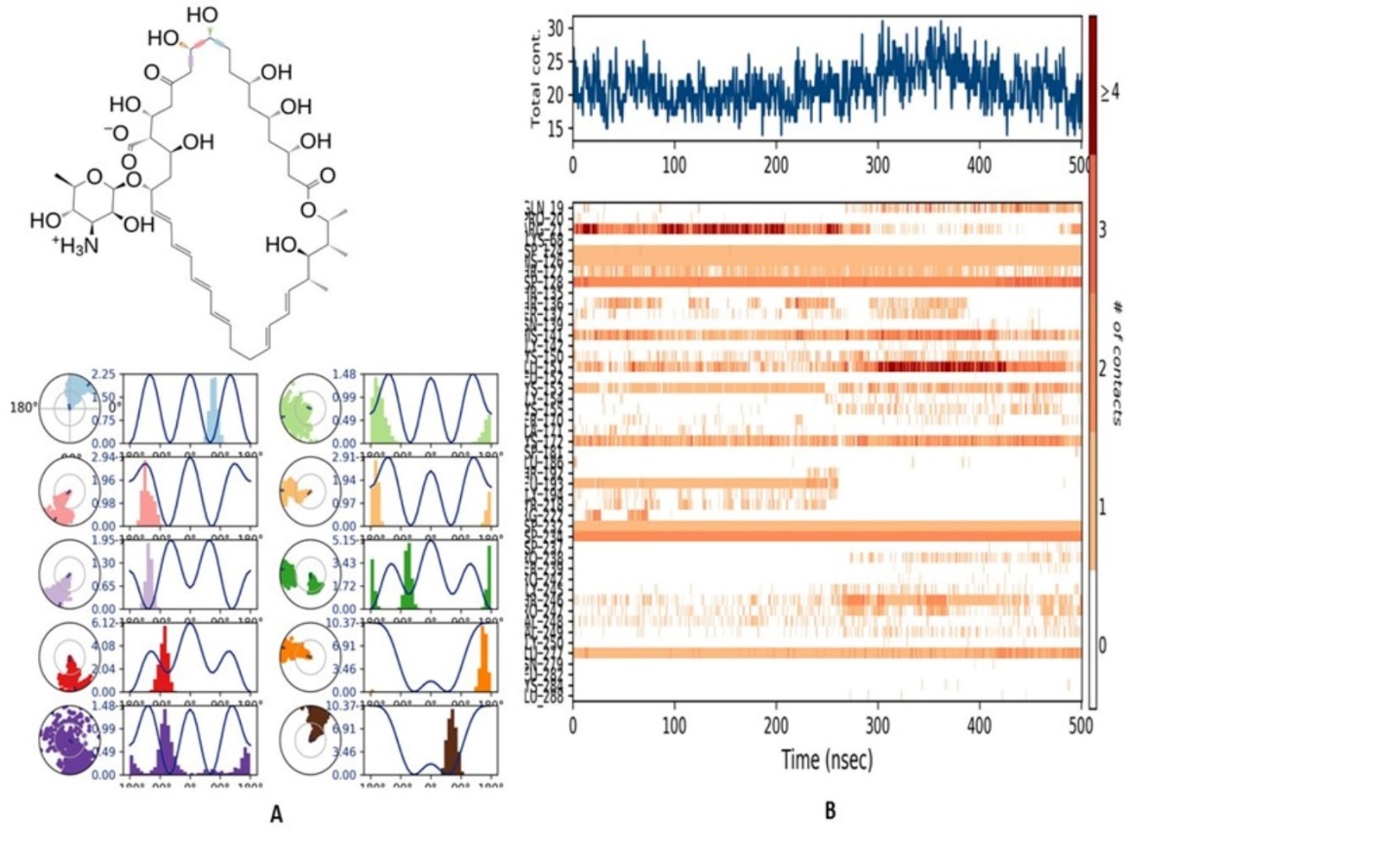


**Figure** **A** Presentation of the ligand torsion profile (**A**) and Time lime representation of protein ligand contacts(**B**) of protonated form of ligand **ZINC000252286875** bound arginase-1 protein.


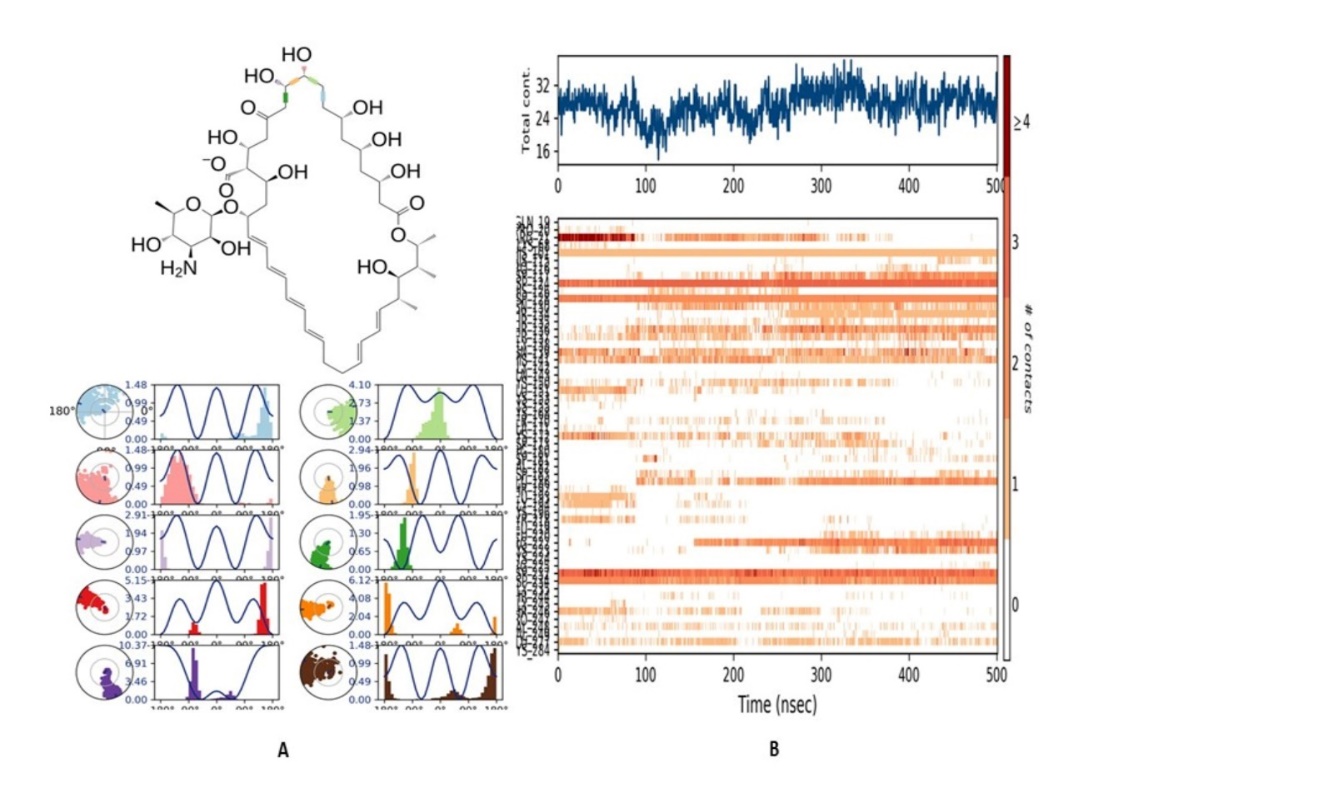


**Figure B** Presentation of the ligand torsion profile (**A**), and Time lime representation of protein ligand contacts (**B**) of non-protonated form of ligand **ZINC000252286875** bound arginase-1 protein.

**
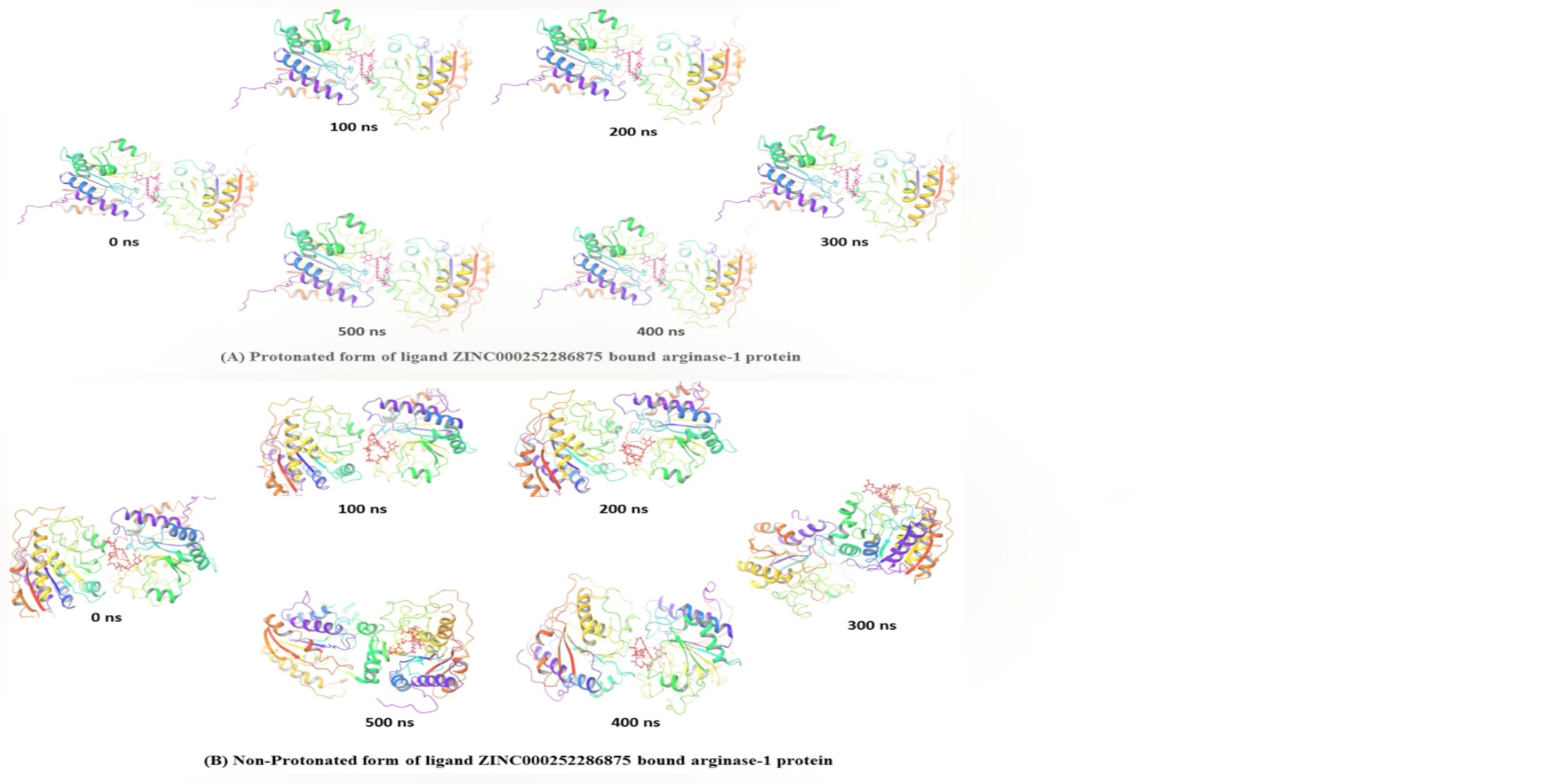
**

**Figure C** Stepwise trajectory analysis for every 100 ns displaying the protein, Arginase-1(PDB I.D: 3KV2) and ligand conformation during 500 ns of simulation of ligand ZINC000252286875 in protonated(**A**), and non-protonated (**B**) forms.


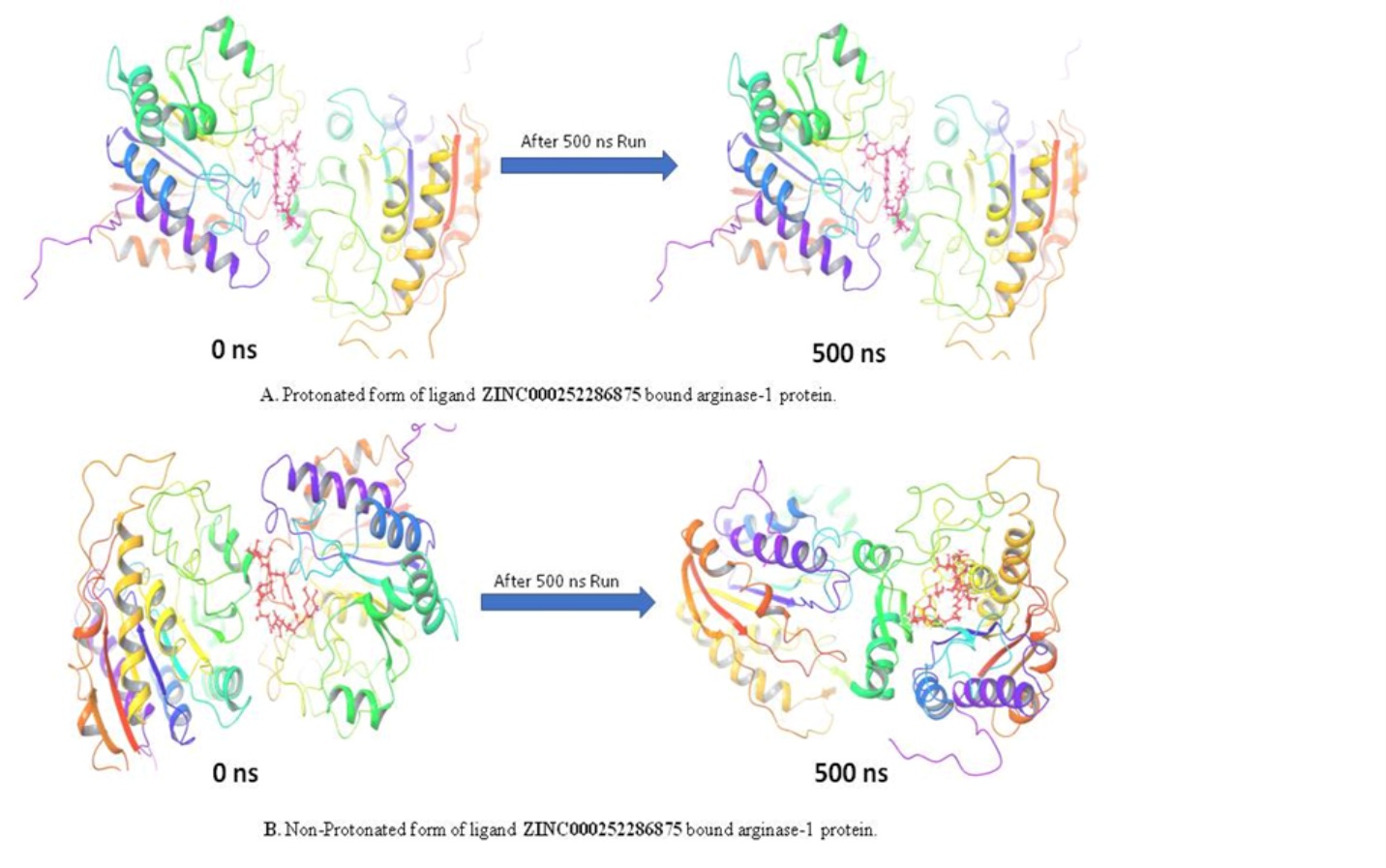


**Figure D** MMGBSA trajectory (0 ns, before simulation and 500 ns, after simulation) exhibited conformational changes of ZINC000252286875 upon binding with the protein 3KV2. In protonated (**A**), and non-protonated forms (**B**).
